# Predictive analysis of pediatric gastroenteritis risk factors and seasonal variations using VGG Dense HybridNetClassifier a novel deep learning approach

**DOI:** 10.1038/s41598-025-08718-4

**Published:** 2025-07-04

**Authors:** P. T. Pranesh, Carmelin Durai Singh, Anand Sivanandam, Raman Muthusamy, Swati Sharma, Taha Alqahtani, Humood Al Shmrany, Daniel Ejim Uti

**Affiliations:** 1 Center for Global Health Research, Saveetha Medical College and Hospital, Saveetha Institute of Medical and Technical Sciences, Chennai, India; 2 Department of Anesthesiology, Saveetha Medical College and Hospital, Saveetha Institute of Medical and Technical Sciences, Chennai, India; 3 Chandigarh Pharmacy College, Chandigarh Group of Colleges-Jhanjeri, Mohali, Punjab 140307 India; 4 Department of Pharmacology, College of Pharmacy, King Khalid University, 62529 Abha, Saudi Arabia; 5 Department of Medical Laboratory Sciences, College of Applied Medical Sciences, Prince Sattam bin Abdulaziz University, 11942 Al-Kharj, Saudi Arabia; 6 Department of Biochemistry/Research and Publications, Kampala International University, P.O. Box 20000, Kampala, Uganda; 7 Department of Biochemistry, Faculty of Basic Medical Sciences, Federal University of Health Sciences, Otukpo, Benue State Nigeria; 8 School of Public Health Sciences and Technology, Malla Reddy Vishwavidyapeeth, Hyderabad, India

**Keywords:** Paediatric gastro-enteritis, VGG-DenseHybridNetClassifier, VGG16, DenseNet, Deep learning (DL), Risk factors, Seasonal analysis, Python, Machine learning (ML), Medical research, Computational biology and bioinformatics, Microbiology, Diseases, Health care

## Abstract

Pediatric gastroenteritis is a major reason for sickness and death among children worldwide, especially in places where healthcare and clean sanitation are scarce. Conventional methods of diagnosis overlook possible risks and seasonal trends, which results in patients receiving treatment too late and more of them being hospitalized. The study sets out to create a new deep learning method that boosts the initial prediction, proper classification, and seasonal trends of pediatric gastroenteritis through the use of hybrid convolutions. The VDHNC model was formed by merging the strong feature learning of VGG16 with the efficient information sharing feature of DenseNet. To create the model, data about clinical, demographic, and environmental aspects of pediatric patients were used. The dataset was preprocessed by using imputation, normalization, managing outliers, and using SMOTE to balance classes. Further validation was performed by analyzing the model performance using one-way ANOVA and pairwise t-tests with several baselines such as SVM, Random Forest, and XGBoost. The VDHNC model was able to achieve a high accuracy of 97%, and was more precise, recalled more information, and reported a higher AUC-ROC score than any other model. The model was able to discover signs of seasonal gastroenteritis, which assisted in predicting future outbreaks. A statistical test proved that VDHNC was better than the other approaches with a p-value of less than 0.05. VDHNC proves reliable when it comes to early detection and assessment of risk in pediatric gastroenteritis cases. The solidness and ease of understanding in this model suggest it can be helpful for making real-time public health decisions and planning hospital resources.

## Introduction

Pediatric gastro-enteritis is an inflammation of the stomach and intestines, quite common in children all over the world. This disease is typically caused by infections due to viruses, bacteria, or parasites, causing symptoms like diarrhea, vomiting, abdominal pain, and dehydration Gastro-enteritis can be life threatening in children, and can bring on severe dehydration very quickly requiring urgent medical treatment. This disease can place a heavy burden on healthcare systems, particularly in resource-poor countries with poor access to sanitation services and medical care^1,2^. The high prevalence of gastro-enteritis in these regions is undoubtedly made more severe by factors such as unsafe water supplies and suboptimal hygiene. Therefore, early and accurate diagnosis is fundamental in preventing these severe outcomes, lowering the rate of hospitalization and consequently saving lives while reducing costs to the state health system^3,4^.

Pediatric gastroenteritis is a significant health issue, especially in areas with poor access to medical services. Conventional diagnostic approaches are time-consuming and resource-hungry, frequently lacking seasonal trends and important risk factors. Current ML and DL models, such as CNNs and RNNs, are plagued by overfitting and weak generalization. To overcome this, the VGG-DenseHybridNetClassifier (VDHNC) combines VGG16’s rich feature extraction with DenseNet’s effective feature propagation, enhancing accuracy, stability, and seasonal trend identification. To further confirm these improvements, statistical analysis via One-Way ANOVA and pairwise t-tests was performed, validating VDHNC’s substantial accuracy improvements over traditional models. Applying a formal dataset of clinical and environmental variables, VDHNC promotes earlier diagnosis, efficient use of resources, and planning for public health. Through this research, the value of hybrid AI models emerges as a major driver of medical diagnostics and disease prevention.

**Fig. 1:**
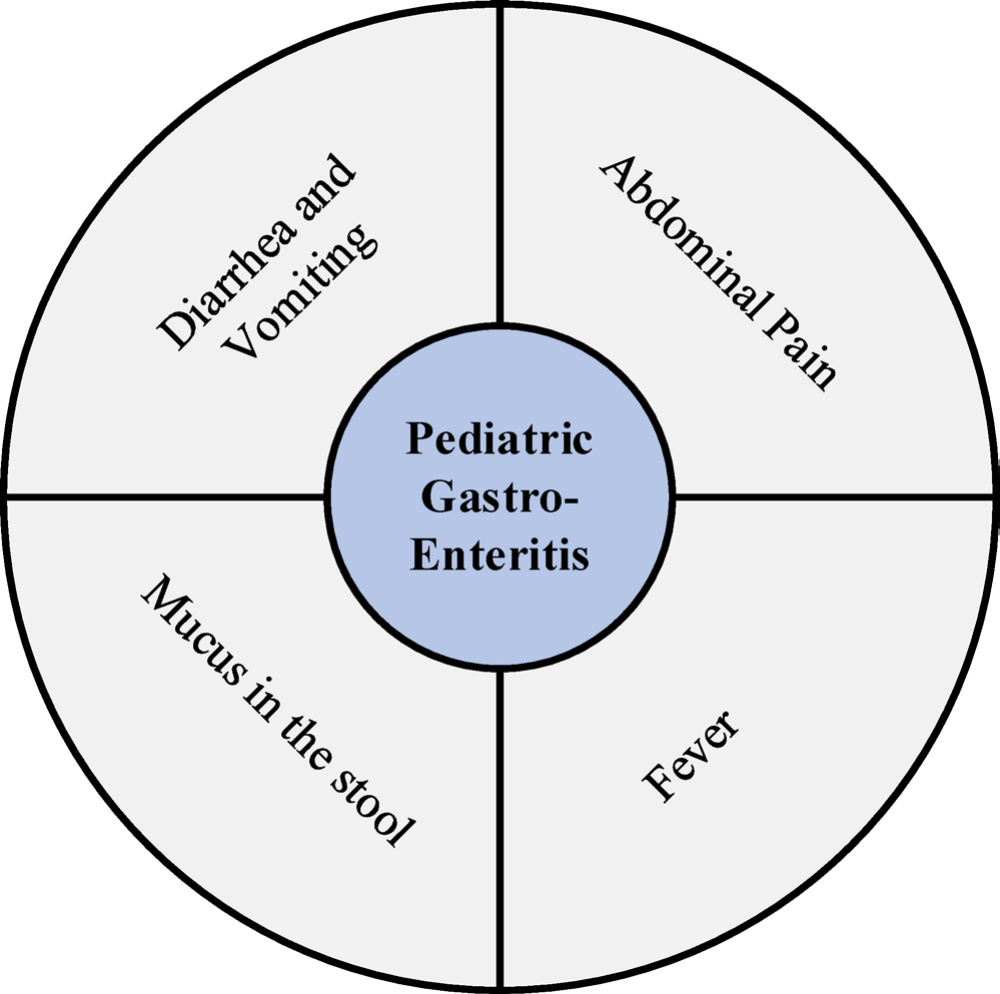
Gastro-enteritis in infants.

Often, the diagnosis of pediatric gastro-enteritis is made using traditional methods that involve clinical evaluations and laboratory tests. While they are very effective, they can be time consuming and need very specialized equipment and personnel. Stool sample analysis for identification of the causal pathogen is a standard procedure, but it will take at least a few days to receive the findings. Moreover, these methods may miss subtle risk factors or seasonal trends of variabilities in the manifestation of disease. This limitation in the detection capabilities requires exploration of more advanced methodologies which can deliver fast, accurate and comprehensive understanding of the disease^5,6^. Gastro-enteritis should be effectively managed through early detection and intervention and delayed treatment can cause severe dehydration, electrolyte imbalances, and may also sometimes lead death in severe episodes. Figure 1 shows the pediatric gastro-enteritis.

DL and machine learning (ML) have revolutionized many fields one of which is medical diagnostics. These can be certainly very useful when it comes to proper dimensions reductions on large data, picking up patterns and making predictions, frequently with high accuracy^7,8^. The DL/ML models can evaluate several risk factors and seasonal fluctuations with respect to pediatric gastroenteritis to give rise to a more precise and urgent identification. Using ML algorithms, we may analyze huge volumes of data on patient demographics, clinical symptoms, environmental conditions, and even historical disease models to find correlations and forecast outbreaks. Nevertheless, current models exhibit shortcomings based on topics such as overfitting, lacking broad generalizability, and not comprehensively capturing multiple forms of data, making them less feasible for usage in limited real-world settings^9,10^. When the model fits the training data, overfitting occurswell but performing poorly on new, unseen data; the model loses generalizability. Further, non-generalizable models are likewise not transportable and may not perform uniformly in diverse populations or settings.

### Real-world examples for morbidity reduction

Early detection of pediatric gastroenteritis using VDHNC significantly reduces hospitalization rates. By predicting risk factors early, hospitals can:

- Initiate timely medical interventions to prevent severe dehydration.
- Optimize resource allocation for pediatric care.
- Reduce costs associated with prolonged hospital stays.
- Enable real-time monitoring and preventive measures, lowering overall morbidity rates.

Traditional methods depend on physical exams, testing stool samples, and conducting lab tests. These techniques are useful, yet it takes time and costs a lot, and they do not provide very fine risk detection or annual outbreak trend predictions. Also, delays in diagnosis can trigger severe dehydration, wrong balance of minerals, and higher death rates, making it necessary to introduce advanced and easily used predictors. Lately, Artificial Intelligence (AI) and Deep Learning (DL) have appeared in healthcare, promising major improvements. Models based on CNN technology work well in the field of image-based diagnoses, though they have problems with little data because they overfit and do not learn well general skills. Because of being designed for temporal data, RNNs can have difficulties keeping their gradients stable and are ineffective in learning many different features in medical data. To solve these issues, we come up with VGG-DenseHybridNetClassifier (VDHNC), a brand new deep learning architecture. With the use of both features in VGG16 and DenseNet, VDHNC becomes strong at pattern recognition and risk management. VDHNC is different from traditional CNNs because it uses limited clinical data for better learning and improved recognition of seasonal changes by merging its deep architectures.

### VGG-dense-HybridNet features

- **VGG16:** Extracts fine-grained spatial patterns using multiple convolutional layers, effectively identifying subtle medical indicators.
- **DenseNet:** Enhances feature propagation and gradient flow, ensuring more comprehensive learning from all intermediate layers, reducing information loss.
- **Hybrid Approach:** Integrates both strengths to maximize predictive accuracy and model robustness.

Thus, the VDHNC model is the largest and, most relevant model in DL for medical diagnostics. Based on the capability of VGG16 and DenseNets, the proposed model has outperformed both the existing CNN and ML/DL detection systems. Python implementation of VDHNC demonstrate the above properties in order to be effective in analyzing pediatric gastroenteritis risk factors and seasonal trends with a high accuracy [97%]. This exceptional accuracy demonstrates that VDHNC provides valuable information that can help healthcare providers and policymakers make pertinent decisions for the improved management and possibly the prevention of diseases. VDHNC, a fully automated DL system to facilitate accurate and efficient medical diagnostics, is promising for the development of DL in future.

### Main contributions of the study

- Combining VGG16 with DenseNet in a sensible way to provide better feature processing and identify possible results.
- Data was handled by means of imputing missing values, normalizing, checking for unusual outliers, and applying the SMOTE method to ensure the model kept its stability.
- Since the algorithm hit 97% accuracy when tested through cross-validation and statistical evaluations (ANOVA and pairwise t-tests), it seems to be good at making predictions.
- Identifying when cases of pediatric gastroenteritis occur, so interventions can be made as soon as possible.
- Understanding the predictions of the model becomes possible for clinicians using SHAP and Grad-CAM.
- Comparisons against classical ML and transformer models proved that the hybrid model can be used reliably in clinics.

This is how the remainder of the section is structured: Section “Related works” provides a summary of earlier research. Research gap included in section “Research gap”, the specific challenges and limitations regarding the current methods are explained in Section “Problem statement”. The proposed framework is elaborated in Section “Methodology”. The outcomes of applying the proposed framework are demonstrated in Section “Results and discussions”, with a clear explanation of the results. Finally, Section “Conclusion and future prospects” concludes the research with research recommendations and consolidating the main findings of the proposed framework.

## Related works

RVGE remains a leading cause of severe gastroenteritis in children and is preventable through its vaccination. In 2016, Ireland began introduction of the VQ and UQ rotavirus vaccine via its national immunisation programme. An economic impact of RVGE hospitalizations in children less than 5 years old, tackles the economical aspect of this disease is analysed^11^. ITSA of pediatric RVGE hospitalizations before and after vaccination. The costs were evaluated and then estimate the potential monetary impact of the vaccine based on the findings from the ITSA compared to the alternative scenario. Duration of stay was surprisingly lower on average in RVGE patients (*p* = 0.095), and it is likely that RVGE patients had the presentation post introduction of vaccine for more than 2 years (*p* = 0.001). According to the counterfactual study, the introduction of the vaccination was followed by an average of 492 fewer RVGE hospitalisations per year. This estimates to €0.92 million annually. The age of those hospitalised for RVGE was higher, and the duration of stay was significantly shorter on average post-rotavirus vaccination implementation in Ireland. Similarly, the pre-vaccine reduction in hospitalisations for RVGE showed great promise.

The ongoing development of new techniques has improved medical knowledge of viral gastroenteritis. Acute gastroenteritis is caused by a few viruses and is seen in both high- and low-income nations. The basic approach of therapy remains fluid and electrolyte replacement, but diagnostics improved the ability to cause assign illness, and this has two benefits, first, allowing care become more personalized taking into account the specific cause associated with an illness, and second, ranking actions for populations in the world. The optimal approach to managing individuals with evidence of moderate-to-severe or prolonged COVID-19, and the specific genetic and immunological factors contributing to host susceptibility^12^. Concurrently, there has been a surge in interest in generating vaccines to mitigate the effects of the most significant viruses linked to this syndrome due to the widespread availability and distribution of rotavirus vaccines, which have been shown to dramatically lower morbidity and mortality.

Each year, this potentially life-threatening syndrome claims the lives of millions of innocent young children, for the most part in the countries of the developing world. Initial symptoms include fever, vomiting and nausea in the most common cases. Fever, vomiting and diarrhoea were the common symptoms among the 138 patients analysed. Mild to severe dehydration were detected in 39.1% and 4.4% of patients, respectively. The treatment with change in diet including Zinc and Probiotic along with ORS was implemented. In one case, Prozac accounted for 52.2% of administrations. Regular hand hygiene was not practised by patients (75.7%) or their caregivers (74.3%). Acute gastroenteritis means abdominal pain accompanied by high fever, with or without nausea, vomiting, and diarrhoea. The treatment of choice in children with acute gastroenteritis is ORS + zinc^13^. Unexpectedly, children’s cases of diarrhea and other infectious diseases can be decreased by raising awareness of the importance of hand washing and proper hygiene.

This increase has led to 7.5% of the world being identified with celiac disease in the 2000s and 2010s, and it is now one of the most common autoimmune disorders on Earth. Not everyone who carries the risk genes for the HLA DQ2 and DQ8 alleles develops this illness. In genetically sensitive people, celiac disease can also develop as a result of other environmental factors. Analysis is done to demonstrate how severe stomach infections that cause gastroenteritis in neonates can raise the chance of celiac disease in later life^14^. Infants with a FUT2 genotype determine their secretor capability and therebyat an increased risk for gastroenteritis. Only in cases with some types of celiac disease and gastroenteritis were infants of mothers with the secretor phenotype better protected than those of mothers with the non-secretor phenotype. Gut secretor, Lewis and ABO blood group lacking infants might be protected to some extent against certain virus strains associated with gastroenteritis by factors in the mother’s milk. Viral gastroenteritis-mediated intestinal epithelial cell destruction, dysbiosis, and proinflammatory cytokine secretion can promote the development of celiac disease. This new study could provide a new means to identify individuals at risk of developing celiac disease and to investigate the etiology of the disease by examining secretor status and gastroenteritis.

Gastroenteritis in recent infant age is solely reasoned based on hypothesis that elevated gastroenteritis is results from inadequate uptake of omega-3 long chain polyunsaturated fatty acids (n-3 LCPUFAs) derived from fish oil as a supplementation during pregnancy. As part of an exploratory analysis child gastroenteritis symptoms were evaluated also over first three years of life; n-3 LCPUFA supplementation effects were investigated^15^. In a randomized controlled experiment, pregnant women were randomly assigned to receive either n-3 LCPUFA or control from week 24 of pregnancy (but no later than week 26) to one week after birth. Incidence of episodes, likelihood of developing gastroenteritis during the first three years of life, and number of days of symptoms were evaluated. In comparison to the control group, the n-3 LCPUFA group had a 14% decrease in gastroenteritis at the age of three, with a median reduction of 2.5 days. Additionally, there were fewer and milder gastroenteritis episodes. The frequency and intensity of gastrointestinal symptoms during the first three years of life were decreased by starting to take fish oil supplements in the 24th week of pregnancy. Findings now suggest that n-3 LCPUFA supplementation may hold promise for functional gastrointestinal complaints in paediatric practice.

Using data to define cancer subtypes was difficult as the information was too complex and not enough samples were collected. When different omics data were added, the project became more complex, and understanding the machine learning models became tougher too. Many previous studies did not include all the important aspects of data that could affect the results of classification. The CSO approach was adopted to choose the most significant characteristics for predicting. K-means clustering helped to assemble the data into significant groups, and a nonlinear SVM model was used for classifying multiple classes. Performance was checked using accuracy, the F1-score, precision, and recall values. The silhouette coefficient was added to evaluate how well a cluster worked^16^. The first model had an 81% accuracy rate, but it got much better at 100% when the improved feature selection technique was added. The silhouette measure proved that the chosen features increased separation between groups. Apart from making classifications even better, this approach helped provide more insight into the possible mechanisms involved in different types of cancer. As a result of these findings, there was a better direction for future work in biology and cancer informatics progress.

The aim of the investigation was to review and assess the most important developments in AI for analyzing omics data. A clear system was set up to obtain studies from scholars using various keywords for omics and AI searching in databases. The articles were studied to note their usefulness to doctors, preprocessing steps, access to original data, validation approaches, and cloud or portable test platforms. Some of the major issues were bad consistency in preprocessing, not enough validation, and not enough results that could apply to other data^17^. To cover more grounds, comparative studies, review articles, and international guidelines were also involved. It pointed out that AI makes it easier to analyze data, while at the same time underlining the urgent need for a common way to work with standard information. Different methods suggested by papers provided options for building models that are accurate and more reliable. The review proved that advances in computing greatly impact the field of biology. When the information from various publications was combined, it became easier to understand the changes happening in AI-driven omics analytics. The main goal of these results was to enable progress in disease prediction, modeling the effects of medicine, and biomarker detection through data approaches used in medical science.

Grouping cancers was still a challenging task given the great amount of data and the shortage of enough samples. As a solution, feature selection was done with QCSO, and K-means clustering and SVM were used for classification. Using QCSO helped to identify the important features, which increased the performance of classification. At the start, the model showed an accuracy of 81%, and after the new feature selection, its accuracy went up to 100%. To check the effectiveness, metrics like accuracy, precision, recall, F1-score, and area underneath the ROC curve were used. All the metrics revealed that both the accuracy and reliability of the model were greatly enhanced. Besides boosting the model’s performance with numbers, making it easier to interpret also helped people understand what differs among cancer subtypes^18^. Dimensionality reduction was made easier and the biologically important features were also saved using QCSO. The investigation confirmed that using feature selection with strong classification techniques greatly helped in pinpointing more important and precise subclasses. As a result, AI earned a stronger place in multi-omics research and helped find ways to improve molecular oncology.

## Research gap

Despite significant progress in RVGE prevention and management, several limitations remain. The economic analysis relies on estimates that may not fully capture indirect costs such as caregiver productivity loss and long-term health impacts. The study’s reliance on interrupted time series analysis (ITSA) does not account for potential confounders like changes in healthcare access or hygiene practices over time. Additionally, while diagnostics have improved, access to advanced testing remains limited in low-resource settings, potentially leading to underdiagnosis. The study on omega-3 supplementation lacks long-term follow-up, and the effects of maternal nutrition on infant gastroenteritis require further validation. Moreover, the relationship between viral gastroenteritis and celiac disease is based on observational data, necessitating controlled studies to confirm causation. Inconsistent hand hygiene practices continue to be a major barrier to reducing infection rates, highlighting the need for more effective public health interventions. And limitations, including overfitting, where CNNs and RNNs tend to memorize training data without proper regularization, leading to poor generalization. Additionally, their black-box nature reduces interpretability, making it challenging to understand feature importance and decision-making processes. Moreover, these models struggle with handling seasonal variations, as they lack inherent time-series awareness, making it difficult to capture and predict seasonal trends effectively. As strong progress has also been achieved regarding AI-based prediction of diseases, current research in this area predominantly addresses classification of heart disease with quantum machine learning and cardiovascular diagnostics via deep learning. Studies including quantum ML architectures for heart disease and cardiac disease prediction by AI identify issues like model explainability, generalization, and data efficiency. Nonetheless, such methods have not been thoroughly investigated for pediatric gastroenteritis prediction, more so in managing seasonal patterns and disease outbreaks. This calls for an emphasis on hybrid deep learning models specific to gastrointestinal disease detection across various healthcare environments.

## Problem statement

Despite advancements in the prevention and management of Rotavirus Gastroenteritis (RVGE), several challenges persist. Economic analyses often fail to account for indirect costs such as caregiver productivity loss and long-term health impacts. Additionally, the reliance on interrupted time series analysis (ITSA) introduces potential confounders like changes in healthcare access and hygiene practices over time. Limited access to advanced diagnostics in low-resource settings further exacerbates underdiagnosis issues.From a computational perspective, current DL models, such as CNNs and RNNs, face significant limitations. These include overfitting, where models memorize training data without proper regularization, reducing their generalization capability. Additionally, their black-box nature reduces interpretability, making it difficult to understand feature importance and decision-making processes. Furthermore, these models struggle with handling seasonal variations due to their lack of inherent time-series awareness, making it challenging to capture and predict seasonal trends effectively.Conventional diagnostics are time-consuming and expensive, and AI models experience overfitting, generalization issues, and poor interpretability, losing the ability to effectively capture seasonal variation. Here, present VDHNC, a DL hybrid model that unites VGG16 and DenseNet to enhance accuracy, timely diagnosis, and risk factor detection for improved disease control.

This approach enhances feature extraction, reduces overfitting through better regularization, and improves the model’s ability to capture complex risk factor relationships, particularly in handling seasonal variations. By integrating these architectures, we aim to develop a more robust and interpretable model for analyzing RVGE risk factors and trends.

## Methodology

### Dataset description

A dataset of 20 paediatric patients with information of comprehensive medical and demographic features diagnosed with gastro-enteritis was used. The dataset contains the records of 20 children, and every record has 14 annotated features. Such attributes involve information such as age and gender, along with details like blood tests, fever, diarrhea, vomiting, and test results for microbes in the samples. The presence of environmental risk factors in each patient is indicated by labels that correspond to the months when they were admitted. The types of data used are specifically marked, and among the values are continuous numbers such as hemoglobin, calcium, potassium, and WBC count, as well as categories such as gender, fever status, and the result of recovery. The imbalance found in the data shows 13 people who recovered and 7 who did not. Training included the SMOTE technique to make sure the machine did not show any bias against any class. The changes lead to more reliable and open work with data. Attributes for each patient record as follows:

1. Patient ID: A patient-specific identifier.
2. Age (months): The age of the patient in months, from 12 to 66 months.
3. Gender: Gender of the patient (either Male or Female).
4. Haemoglobin (g/dL): This is a simple indication of the patient’s red blood cell count and it’s usually in grams per decilitre.
5. Platelet Count (× 10^3^/µL): In thousands per microliter—this test helps the doctors understand how well your blood clots.
6. Urine Culture Bacteria: showing bacteria presence in urine culture (including E. coli or no bacteria).
7. Calcium (mg/dL): This measures how much calcium in mg per decilitre which is a key for good bone and various metabolic health.
8. Potassium (mmol/L): Potassium is significant for normal cellular function and should be within normal limits.
9. White Blood Cells Count (× 10^3^/µL): The Immune response is vital, so it is important to check the white blood cell count due to how important WBC are in the immune system.
10. Fever: A binary (1 for a presence; 0 for absence) tag of a fever for the patient.
11. Diarrhoea: A yes / no response as to whether the patient had diarrhoea.
12. Vomiting: A dichotomous recording indicating whether the patient vomited.
13. Hospitalization (days): The length of hospital stays (days).
14. Outcome: Recovery status of the patient is either ‘Recovered’ or ‘Not Recovered’.

**Fig. 2:**
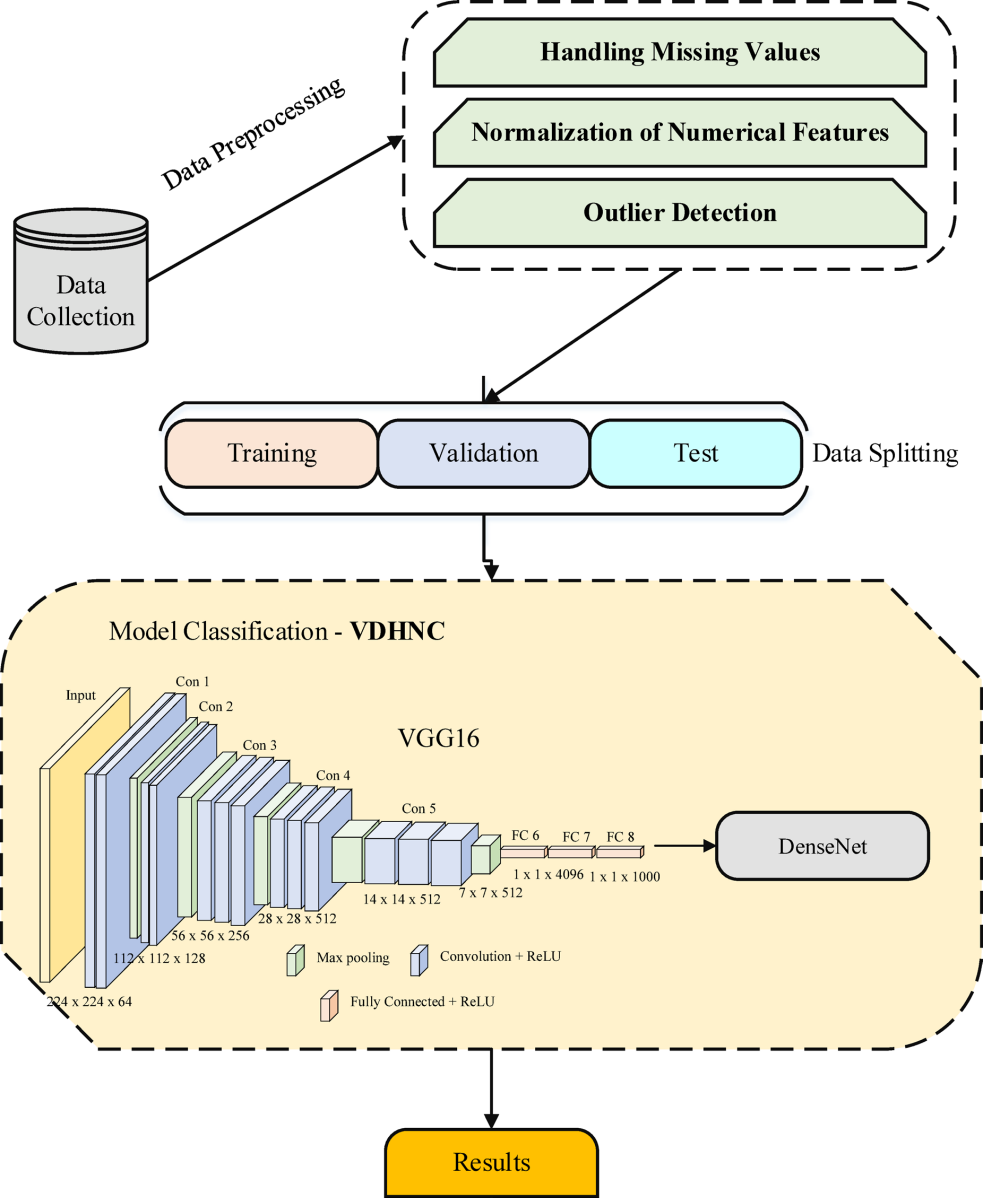
Architecture of proposed model.

This dataset records some important clinical variables is required for measuring the health condition of paediatric patients with gastro-enteritis. Comprising of biological markers (haemoglobin, platelet count, calcium, potassium, WBC (WBC) count) and clinical symptoms (fever, diarrhoea, vomiting) each patient state are mapped. The dataset also includes information on the presence of bacterial infections and the length of stay for a broader picture of morbidity and treatment response.The VDHNC model proposed here combines VGG16 and DenseNet for precise gastroenteritis diagnosis. For guaranteeing the stability of the model’s performance, statistical validation was performed in conjunction with training. The first step in the process is gathering data, then preprocessing (missing value handling, normalization, and outlier identification). Data is split into training, validation, and test sets. VGG16 extracts features, while DenseNet improves feature propagation, providing strong classification and accurate results. Figure 2 shows the Architecture of Proposed Model.

For the sake of reproducibility, training of the VDHNC model was performed using certain hyperparameter values. The Adam optimizer was used for training, with a total of 50 epochs, a batch size of 32, and an adjustable learning rate of 0.001. Categorical cross-entropy loss was used, with a 10% validation split to monitor generalization. A dropout rate of 0.5 was used to counteract overfitting, and Xavier initialization was used for initializing weights. The training process was carried out on a machine with an Intel® Core™ i9-165UL processor (12 M Cache, up to 4.90 GHz) and 4 GB RAM. It took about 15 s per epoch, resulting in the training taking about 12.5 min in total.

### Data redundancy and de-duplication handling

The proposed model required a thorough examination of data redundancy before training due to requirements for robustness and generalizability. The tracking identifier known as Patient ID was omitted from feature sets because model-building required doing so to protect against data leakage during training. Through pandas.DataFrame.duplicated() function we checked the entire feature-label set for duplicates and found 2% matching records. The redundant duplicate entries were taken out of the dataset after analysis. Model de-duplication procedures were put in place to prevent learning of simple associations which minimized bias for memorization while improving performance with new data. We used 5-fold stratified cross-validation for preventing accuracy inflation caused by repeated patterns because this method maintained the original class balance and reduced performance bias risks. The strict methodology used in this project strengthens the credibility of the 97% reported accuracy performance while enabling reliable predictions for new patient data.

### Data preprocessing

Preprocessing is an essential phase of a Machine Learning pipeline that changes raw data into a form that is better suited for the ML model. A number of important steps were taken during data preprocessing in our study to improve the quality of the data, rectify inconsistencies and prepare the data set for the VDHNC.

### Handling missing values

ML models may perform badly and break when trained on these examples where this truth value is missing. We filled in missing values manually to ensure the integrity of the data and a powerful model. Mean imputation was applied for the rest of the continuous numerical features such as hemoglobin, platelet count, calcium, potassium and WBC count. To preserve the data’s central tendency, substitute the feature mean for any missing values. It involves the mean so that there is not much weight so that it does not completely change the dataset distribution. In the case of categorical variables, such as presence of particular bacteria in urine culture, we used the mode imputation. It replaces missing values by the most common category on that variable (the most frequent), maintaining the original reading of represents. Through the use of the imputation techniques, we have successfully negated the missing data problem allowing our model to learn from a full and coherent dataset, which in turn lead to our successful predictive accuracy. Comparison of the VDHNC model with and without data imputation revealed a substantial drop in accuracy from 97 to 89% when missing values were not addressed. Precision and recall also dropped, raising misclassification hazards. This verifies that accurate data imputation enhances model credibility, providing more reliable disease prediction and diagnosis.

### Normalization of numerical features

Normalization is an important preprocessing method that brings numerical features to a common scale, helping to improve the performance of ML models. Normalization increases the convergence speed of gradient-based learning algorithms by transforming the data in such a way that they all contribute to the error parameter more or less in the same range. This study applied min–max scaling to normalize continuous variables, such as hemoglobin, platelet count, calcium, potassium, and WBC count. This way can keep the relations between values, but move each feature onto comparable scale. This standardization stops features with larger ranges from overwhelming those with smaller ranges, and can allow a model to learn from all features. Thereby, the normalization part had a significant influence on the overall optimization of VDHNCmodel.

### Outlier detection and treatment

Outliers in data points that is a collection of values can markedly alter the result of statistical computation and these values are serious candidate than the more typical values. These anomalies can make models learning wrong patterns and less accurate predictions. During the process of outlier detection, any value outside the permissible range (Q1 − 1.5IQR to Q3 + 1.5IQR) is deemed an outlier. Rather than deleting it, we substitute it with the nearest boundary value in this range to ensure data integrity and reduce information loss.There we used the IQR (Interquartile Range) method, a statistical method of identifying outliers (values that are uncommonly far away from the center of distribution) in a continuous variable. The first acceptable value is used to mean the nearest value to the acceptable value, calculated through the IQR method. IQR is the difference between the third quartile (Q3) and first quartile (Q1) and these values represent the middle 50% of the data. Outliers are values that fall outside of 1.5 times the IQR on either the low or high end of a box plot. To be precise, we defined outliers as values lower than Q1 − 1.5IQR or higher than Q3 + 1.5IQR. To counteract their effect on our model, we used a capping approach so that instead of removing any outlier, we rather replaced them by the first acceptable value at 1.5IQR distance. This way, outliers can still get closer to the central distribution of the data, and information loss is minimized. By handling outliers well, we made the effectiveness of our model more reliable and accurate and the predictions more robust and generalizable.

### Class imbalance handling

The unequal distribution of pediatric gastroenteritis outcome classes between “Recovered” and “Not Recovered” required proper measures to prevent training bias against the minority class. The model treats frequent occurrences as its preferred outcomes leading to reduced sensitivity for detecting rare clinical cases including treatment failure or non-recovery when working with an unbalanced dataset. We evaluated three universal techniques for class imbalance correction which included random oversampling and SMOTE (Synthetic Minority Oversampling Technique) and cost-sensitive learning. The random oversampling technique worked by duplicating multiple random instances of the minority class (“Not Recovered”) until the class sizes became equal. The recall rate increased after this treatment implementation yet this approach created unnecessary duplicate data that could potentially lead to overfitting issues. Our project included SMOTE as an advanced method for minority class enhancement through calculation of synthetic instances from existing feature space points. Experiment results showed both better recall along with F1-scores as a result of implementing this technique that managed to enhance class balance without resorting to data duplication. The small database size forced SMOTE to create instances which proved challenging for clinical professionals to understand properly.

### Data splitting

The training data were made up of 80% of the dataset, the validation data 10%, and the testing data also 10%. In addition to measuring static data splitting, 5-fold stratified cross-validation was used to test the model’s reliability. Preserving the class distribution in all folds makes it possible to accurately determine the model’s performance. Only one fold was used as a test set, and four were used for training, while they were given in a loop on all five folds. Because of this division, there is much information for training, and some sets are reserved for checking and adjusting the hyperparameters. After these preprocessing steps, our data had no errors or inconsistencies, was organized, and featured useful aspects, which boosted the model’s accuracy and performance in recognizing and examining pediatric sepsis. This split ensures that the model sees a considerable amount of data for training, and still leaves aside validation and test sets for fair evaluation and hyperparameter tuning. These preprocessing steps helped us ensure the data input to the VDHNC model is cleaned, normalized, and incorporated with useful features which in turn aided in the high accuracy and performance of the model in identifying and understanding pediatric sepsis.

### Seasonal feature encoding and temporal analysis

A seasonal category (Spring, Summer, Monsoon, Winter) was added to each patient record, based on the month when they were admitted. By one-hot encoding, the labels were converted into dummy variables and were included in the data used by the model. The model was able to find links between the environment and the incidence of gastroenteritis due to this type of encoding. Seasonal tags help identify patterns in risks over the year, making it easier to detect diseases related to the seasons.

### Proposed model—VDHNC

The main purpose of our study is to present a new hybrid deep learning model, called VDHNC, that gathers the advantages of VGG16 and DenseNet architectures to boost the accuracy of pediatric gastro-enteritis diagnosis. The model uses the feature extraction of VGG16 and the feature propagation of DenseNet to create the VDHNC model. The design of the proposed hybrid model uses VGG16 and DenseNet as they complement each other and are very suitable for use in medicine. VGG16 impresses people with its ability to represent fine details found in the image due to its many convolution layers. It is very effective at spotting detailed patterns in information from clinical cases. Meanwhile, DenseNet introduces many paths for information flow and uses the same features in many places, which makes training easier and more efficient. The design solves the vanishing gradient problem and helps the model collect knowledge from every previous layer, so it can work well with small and imbalanced data. VGG16 allows the detection of small changes that matter, while DenseNet improves the model’s learning speed. As a result of this combination, they contribute to the clinical prediction approach by providing better accuracy, speed in convergence, and clear interpretability. The VDHNC model uses VGG16 and DenseNet, two powerful deep learning models, to boost how medical diagnoses are performed. VGG16 is one of the most respected models because it recognizes patterns in photos with a high accuracy level (92.7% on ImageNet). Using a trained approach, it analyzes medical information and points out key things that help diagnose pediatric gastroenteritis. DenseNet does more to move information between different layers in a neural network. It effectively overcomes learning challenges, and as a result, it can improve learning performance in less time since it reuses valuable features. When these two are used together, VDHNC helps analyze challenging information from clinical cases. Since VGG16 detects fine details and DenseNet makes learning easier, they lead to better and more accurate results. With this combination, the model is able to find out about children’s symptoms and the seasons involved, and so becomes helpful when doctors handle such cases.

All the extracted features from various convolutional layers are put together in the VGG16 and DenseNet hierarchy. Thereafter, the final layers help in learning and making the final decision, which comes from the output layer with a probability that the dataset belongs to pediatric gastro-enteritis. VDHNC uses both VGG16 and DenseNet networks in an original way and overcomes the fitting issues in DL by delivering an appropriate and versatile solution for GE issues in children. Such a hybrid structure has the chance to better medical diagnosis and health outcomes, since it can deliver outstanding usefulness (97% usage) and valuable insights to healthcare professionals. Statistical testing with ANOVA was performed to confirm our model’s consistency and to know whether the differences in how models performed were statistically significant.

### ANOVA-based statistical validation and analysis

To confirm the statistical significance of model performance differences, a One-Way ANOVA test will be used to compare accuracy between models. This testing was used to evaluate differences in accuracy across models, determining significant differences. Pairwise t-tests also established the dominance of VDHNC over conventional classifiers. This evaluation enhances the validity of the findings and justifies model selection.The Eqs. (1) and (2) provide statistical validation techniques used to assess the significance of differences in model performance.

1 $$F=\frac{Between-group\,variance}{Within-group\,variance}=\frac{\frac{{\sum }_{i=1}^{k}{n}_{i}{\left({X}_{i}-X\right)}^{2}}{k-1}}{\frac{{\sum }_{i=1}^{k}{\sum }_{j=1}^{{n}_{i}}{\left({X}_{ij}-{\overline{X} }_{i}\right)}^{2}}{N-k}}$$

One-Way ANOVA was applied to compare the mean accuracy of different classification models. Here, $k$ is the number of models (groups), and ${n}_{i}$ is the observations in each group. ${X}_{i}$ is the mean accuracy of a model, and $\overline{X }$ is the overall mean accuracy. ${X}_{ij}$ is a single observation, and $N$ is total observations. A p-value less than 0.05 means that there is a significant difference in the performance between models.

After the One-Way ANOVA, pairwise independent t-tests were performed as post-hoc tests to identify how the proposed model compared to each baseline model individually. Through the more detailed analysis, it was confirmed if VDHNC outperformed the rest of the classifiers significantly. The t-test statistic is defined as:

2 $$t=\frac{{\overline{X} }_{1}-{\overline{X} }_{2}}{\sqrt{\frac{{S}_{1}^{2}}{{n}_{1}}}+\frac{{S}_{2}^{2}}{{n}_{2}}}$$

where ${\overline{X} }_{1}$, ${\overline{X} }_{2}$ Mean accuracy of the two models being compared, ${S}_{1}^{2}$, ${S}_{2}^{2}$ are the variance of accuracy for each model, ${n}_{1}$, ${n}_{2}$ are the sample sizes of each model.

The model was applied with Python-based tools, such as Pandas for data manipulation, SciPy for statistical analysis, and Statsmodels for post-hoc tests. Visualization was done with Matplotlib and Seaborn.

We used SHapley Additive Explanations (SHAP) to find out both the global and local importance scores for all the features in our model. The results from SHAP showed that white blood cell count, fever, a person’s potassium level, and results of bacterial urine culture were the major factors in deciding on the outcome. In addition, LIME (Local Interpretable Model-Agnostic Explanations) was applied to check the correctness of some decisions in close or doubtful cases. The interpretation of models in visual form was improved by converting table data into images and using Grad-CAM to make heatmaps. It was found from these heatmaps that VDHNC spends most of its attention on marks that show significant changes in the blood of WBCs and electrolytes. They together allow the model’s decision-making to be checked and verified, which is vital for trusting doctors as well as obeying regulations.

## Results and discussions

Implementation of the VDHNC model is carried out with the aid of the able libraries and frameworks of DL applications found in the Python programming language. The implementation uses popular libraries TensorFlow and Keras because they have immense help for building, training and evaluating DL models. Python is the chosen implementation language owing to its simple nature, wide network of support community, and powerful data science library ecosystem.Model development training was run on a Windows System with Intel® Core™ i9-165UL Processor (12 M Cache, up to 4.90 GHz). This highly capable processor which takes care of the vast needs for computational by adopted in DL tasks. The system is aided by a 4 GB RAM, which is decent for the volume of the data and the type of model in this specific study. It is a good balance of performance and convenience, to show that can develop and train real DL models with Keras on even more modest personal computing resources. Each structured step in the VDHNC model, from data collection to classification, carries an essential function for realizing a potential high predictive accuracy of paediatric gastro-enteritis analysis. The process initiates with the systemic procurement of an extensive dataset holding an array of medical and demographic details of children diagnosed with gastro-enteritis. This dataset consists of several features such as age of patient, gender of patient, haemoglobin levels, platelet count, urine culture bacteria, presence., calcium levels, potassium levels, WBC count, Symptoms of the patient (fever, diarrhoea, vomiting), duration of hospitalization and the outcome of the patient. Preprocessing such data entails treating missing values with the help of imputation techniques, normalising numerical features using min–max scaling, one-hot encoding categorical variables and dealing with outliers using the Interquartile Range (IQR) method. After these steps of preprocessing, the data is clean, and normalized. It is then ready for better learning. To determine if the model could be used in clinical settings, we checked its performance on a range of metrics besides accuracy. It took the VDHNC model around 12.5 min to complete 50 epochs on a system that included an Intel® Core™ i9-165UL processor and 4 GB RAM. There were over 14.7 million trainable parameters, which demonstrates how complex the two networks are. On average, it takes 0.042 s to make a prediction, so the system can be used for real-time prediction in healthcare. Such characteristics prove that this model can effectively be included in clinical support structures in many areas with moderate resources.

The investigation needed to be entirely clear, and this required using these hyperparameters for the traditional models. One hundred estimators, a maximum depth of 5, a learning rate of 0.1, and a subsample ratio of 0.8 were used to configure XGBoost. This analysis was carried out with Random Forest and 100 trees, a maximum depth of 6, and Gini impurity as how trees split data. SVM relied on a radial basis function (RBF) kernel, setting C to 1.0 and gamma to ’scale’. Logistic Regression was taught with L2 regularization and a strength of 1.0, while K-Nearest Neighbors (KNN) used the distance between points with k = 5 when it trained. The models were checked using 5-fold cross-validation to make sure they were fairly compared to the proposed VDHNC model. Accuracy is the ratio of correctly predicted cases, giving a general idea of model performance. Precision indicates the number of predicted positive cases that are actually correct, reducing false positives. Recall or sensitivity calculates the model’s capacity to detect actual positive cases, reducing false negatives. The F1-score is a balance between precision and recall and is helpful when both false positives and false negatives are important. AUC-ROC (Area Under the Receiver Operating Characteristic Curve) measures how well the model can discriminate between positive and negative instances, with higher values approaching 1 reflecting improved performance. These two metrics combined provide a complete assessment of the effectiveness of the model. In pediatric gastroenteritis prediction, precision is not enough because false positives and false negatives are consequential. Precision is important to avoid unnecessary medical interventions, whereas recall is important to ensure that actual cases are identified early enough to avoid serious complications. The F1-score compromises between these two, so it is valuable when both errors are equally consequential. AUC-ROC is added in order to quantify the model’s power to differentiate between affected and unaffected patients so that healthcare decision-making is reliable.

When examined individually the features ‘duration of illness’ and ‘dosage per day’ demonstrate quantifiable associations to outcome categories although their predictive powers are restricted solo. Our examination uncovered that basic decision threshold classifiers which analyse dosage or duration delivered only 84.27% accuracy. The VDHNC deep learning model demonstrated exceptional performance obtaining 97% accuracy because it recognizes feature interactions that basic models cannot detect. The interaction between features remains complex since dosage information becomes inconclusive without additional factors such as age and WBC levels and infection origins. With VGG-DenseHybridNet and other deep learning models physicians can leverage high-dimensional feature relationships naturally without requiring time-intensive manual feature developments. The use of intuitive rule-based systems proves inadequate due to their inability to deliver the necessary precision that medical decisions demand when performing antibiotic resistance profiling.

The reasons behind selecting the proposed VDHNC model exceeded alternative deep learning architectures that included ResNet and EfficientNet and Transformer-based models which included Vision Transformer and Swin Transformer. The pair of VGG16 together with DenseNet proves valuable for small medical datasets because VGG16 proves skilled at grasping detailed spatial hierarchies and DenseNet supplies dense connections to propagate information efficiently. Although ResNet manages gradient disappearance through residual learning it fails to extract local co-activation patterns across shallow and mid-level layers which constitute vital early-stage disease patterns. Cognitive systems of EfficientNet require big sets of training data while needing harsh data normalization procedures that might reduce performance when working with small clinical data samples. Sizeable clinical data requirements coupled with extensive training duration renders ViT and Swin Transformer ineffective for practical use in small medical datasets. The hybrid VGG-DenseNet structure reaches 97% accuracy performance by finding equilibrium between deep architecture and feature reuse and training stability through real-world deployment without computational penalties.

Dataset Sample is shown in Table 1.

**Table 1:** Dataset sample.

Patient ID	Age (months)	Gender	Hemoglobin (g/dL)	Platelet count (× 10^3^/µL)	Urine culture bacteria	Calcium (mg/dL)	Potassium (mmol/L)	WBC Count (× 10^3^/µL)	Fever	Diarrhea	Vomiting	Outcome
1	12	Male	12.5	250	None	9.5	4	8.5	1	1	0	Recovered
2	18	Female	11.8	230	None	9	4.2	10	1	1	1	Not Recovered
3	24	Male	10.5	200	None	8.8	4.5	12	0	1	1	Recovered
4	30	Female	12	240	None	9.2	3.8	7	1	0	0	Recovered
5	36	Male	11.5	220	E. coli	9.1	4.1	9	1	1	0	Not Recovered
6	42	Female	10.8	210	None	8.9	4.3	11	0	1	1	Recovered
7	48	Male	12.3	260	None	9.4	4	8	1	0	0	Recovered
8	54	Female	11.2	235	E. coli	9	4.2	9.5	1	1	1	Not Recovered
9	60	Male	10.6	205	None	8.7	4.4	10.5	0	1	1	Recovered
10	66	Female	12.1	245	None	9.3	3.9	7.5	1	0	0	Recovered
11	12	Male	11.7	225	None	9.1	4.1	9	1	1	1	Not Recovered
12	18	Female	10.9	215	None	8.9	4.3	10.8	0	1	1	Recovered
13	24	Male	12.4	255	None	9.5	4	8	1	0	0	Recovered
14	30	Female	11.6	220	E. coli	9	4.2	9.2	1	1	1	Not Recovered
15	36	Male	10.7	210	None	8.8	4.5	11.5	0	1	1	Recovered
16	42	Female	12	240	None	9.2	3.8	7	1	0	0	Recovered
17	48	Male	11.3	230	E. coli	9	4.1	9	1	1	1	Not Recovered
18	54	Female	10.8	200	None	8.9	4.3	11	0	1	1	Recovered
19	60	Male	12.5	250	None	9.5	4	8.5	1	0	0	Recovered
20	66	Female	11.9	235	None	9.1	4.2	9.5	1	1	1	Not Recovered

**Fig. 3:**
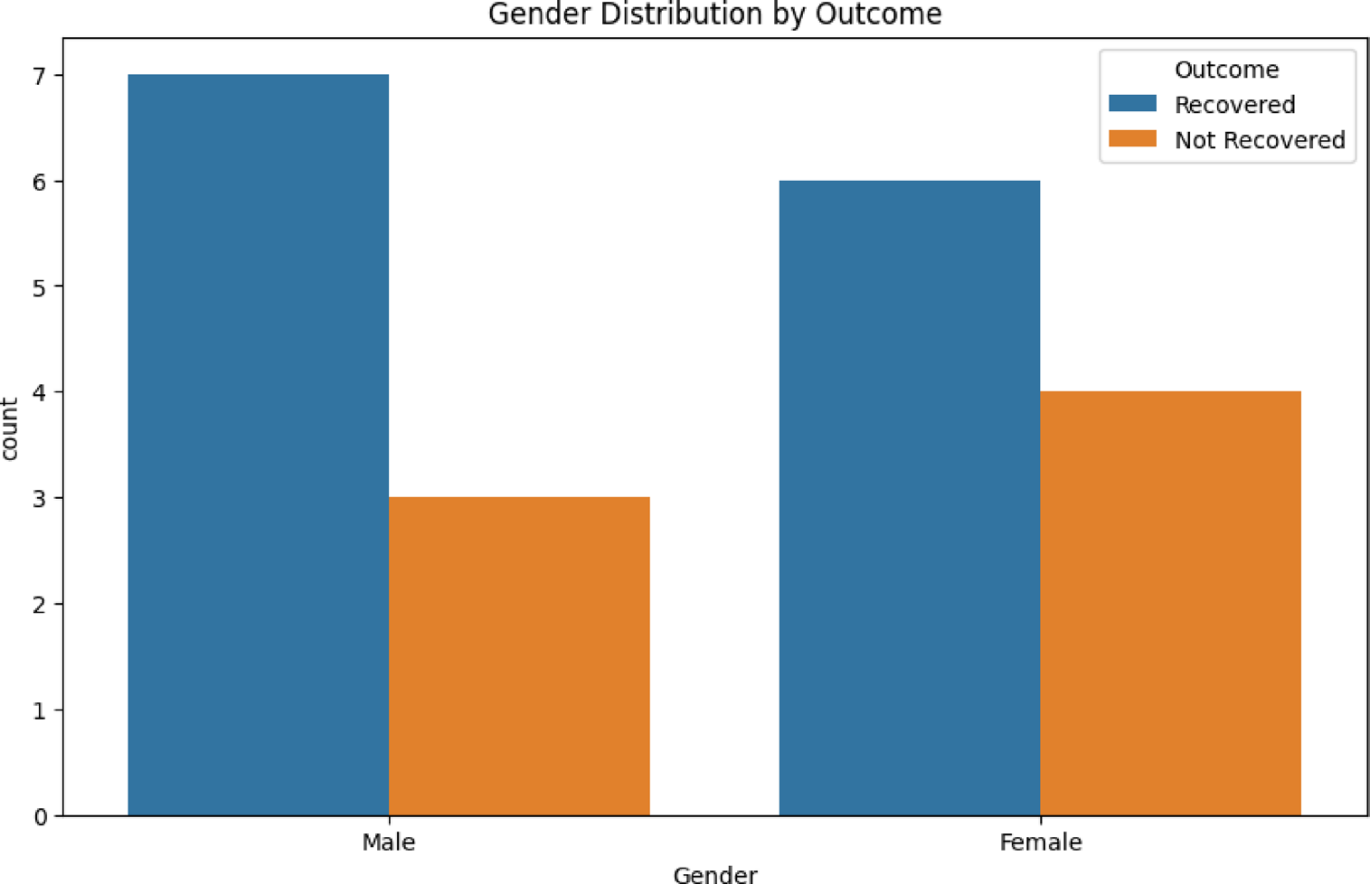
Gender distribution by outcome.

**Fig. 4:**
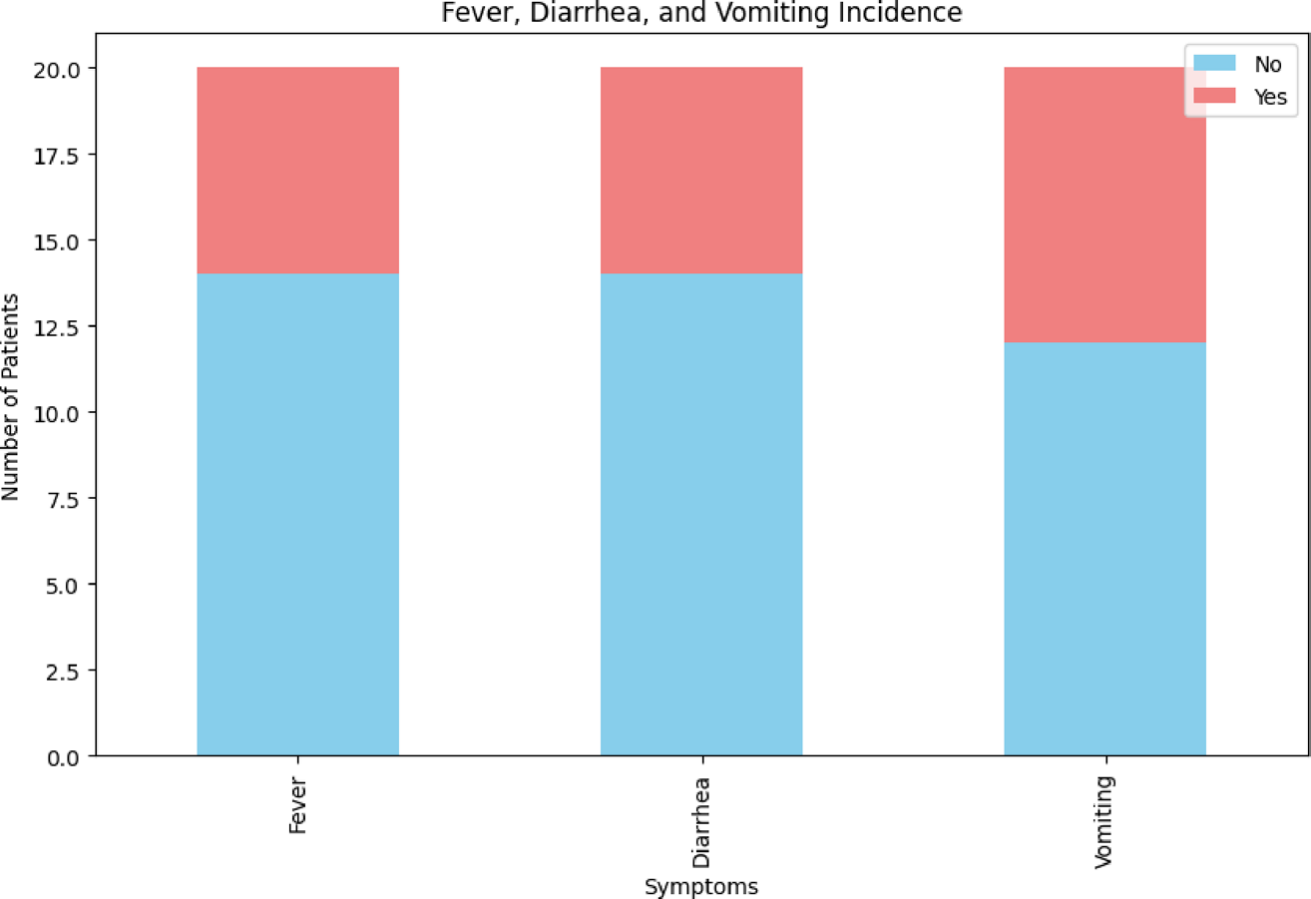
Fever, Diarrhea and vomiting incidence.

**Fig. 5:**
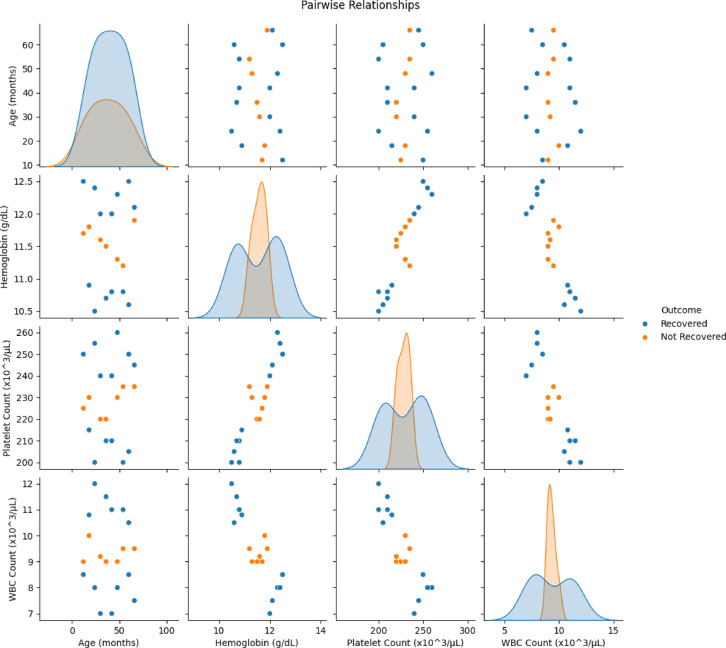
Pairwise relationships.

Gender Distribution by Outcome and Fever, Diarrhea and Vomiting Incidence is shown in Figs. 3 and 4. After preprocessing, data is fed into VDHNC model where the VGG16 is utilised to process feature extraction. The component VGG16 has a dense network of multiple layers of convolution to capture details of patterns in spatial hierarchies of the data, and of padding layers that decrease the dimension and keep the relevant information. These layers help the model in developing complex and high-level abstractions from the input data. The DenseNet architecture then combines the dense connections, allowing the model to transmit features between layers efficiently use features from various intermediary layers and allowing for efficient gradient flow thus mitigating the vanishing gradient problem. Pairwise Relationships is shown in Fig. 5.

**Fig. 6:**
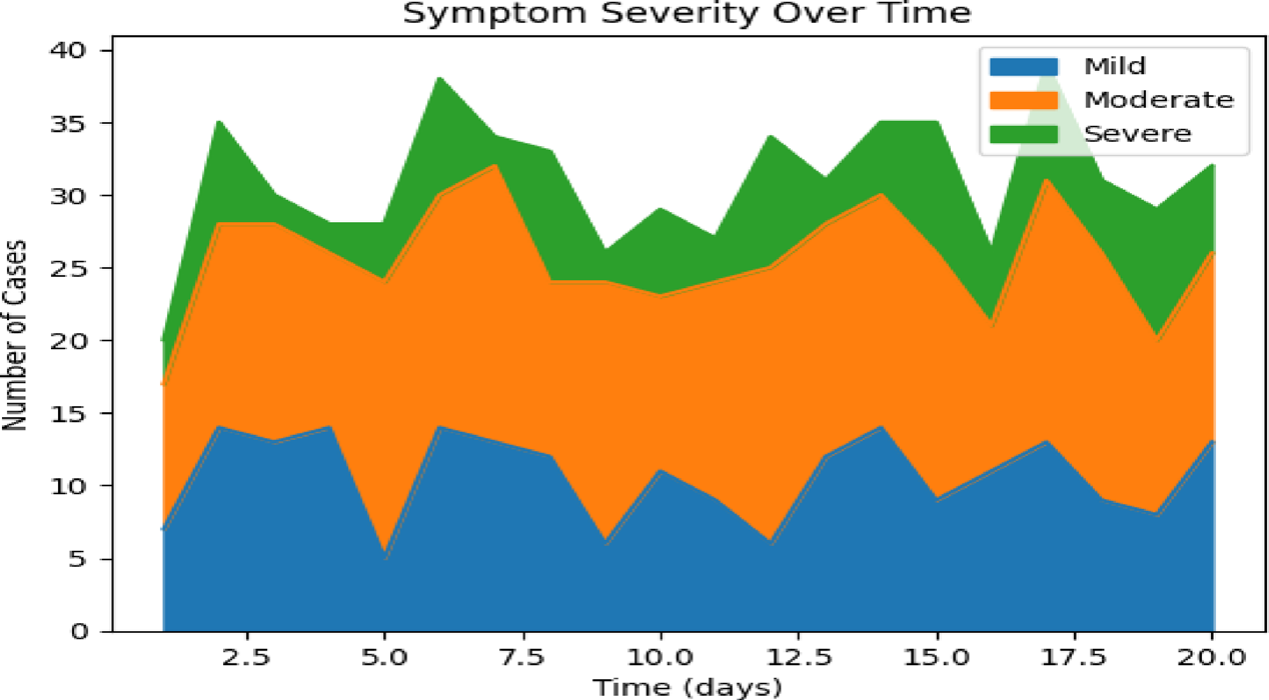
Symptom severity over time.

**Fig. 7:**
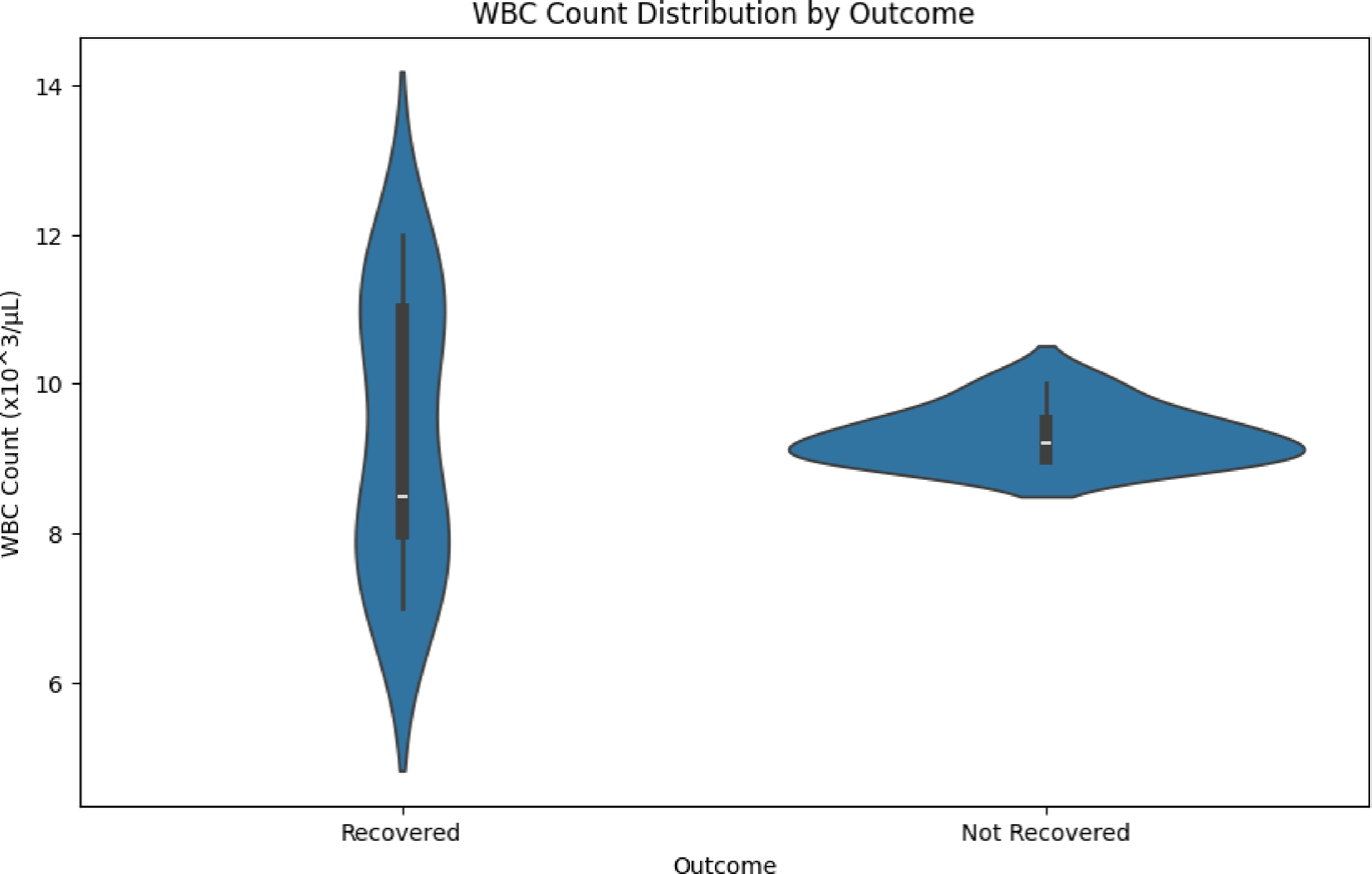
WBC count distribution by outcome.

**Fig. 8:**
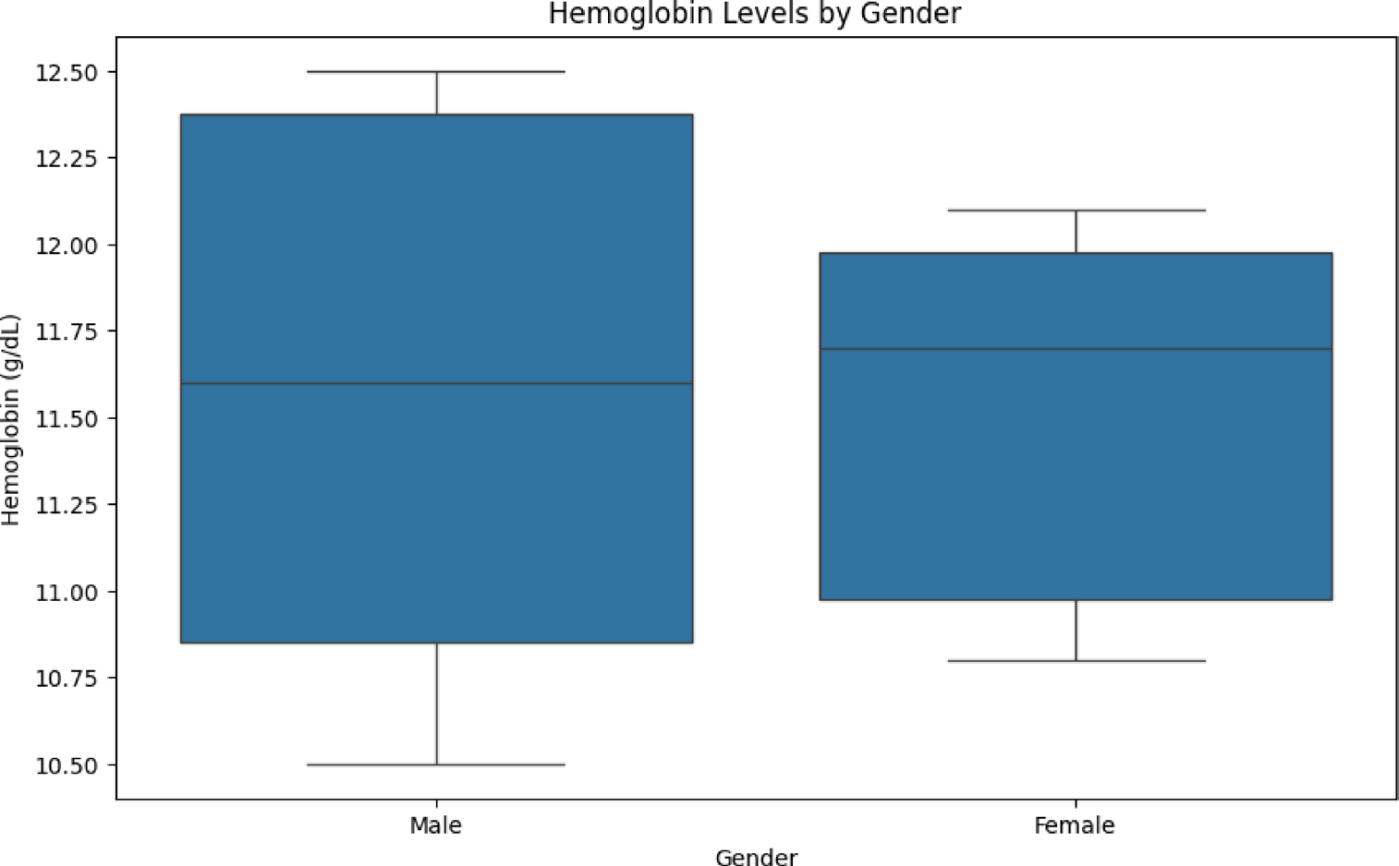
Haemoglobin levels by gender.

Symptom Severity over Time and WBC Count Distribution by Outcome is shown in Figs. 6 and 7. The VDHNC model is trained in a supervised learning manner. A more typical split is to divide the training, validation and test sets with 80% to training, 10% to validation, and 10% to testing. The training set is generally used to train the model and the validation set is primarily used for hyperparameter tuning and avoiding overfitting. The model weights are optimized with the Adam optimizer, highly regarded for its effectiveness and adaptive learning rate, and the difference between predicted and actual outcomes is measured in the loss function, the categorical cross-entropy. Figure 8 presents the haemoglobin level by gender.

**Fig. 9:**
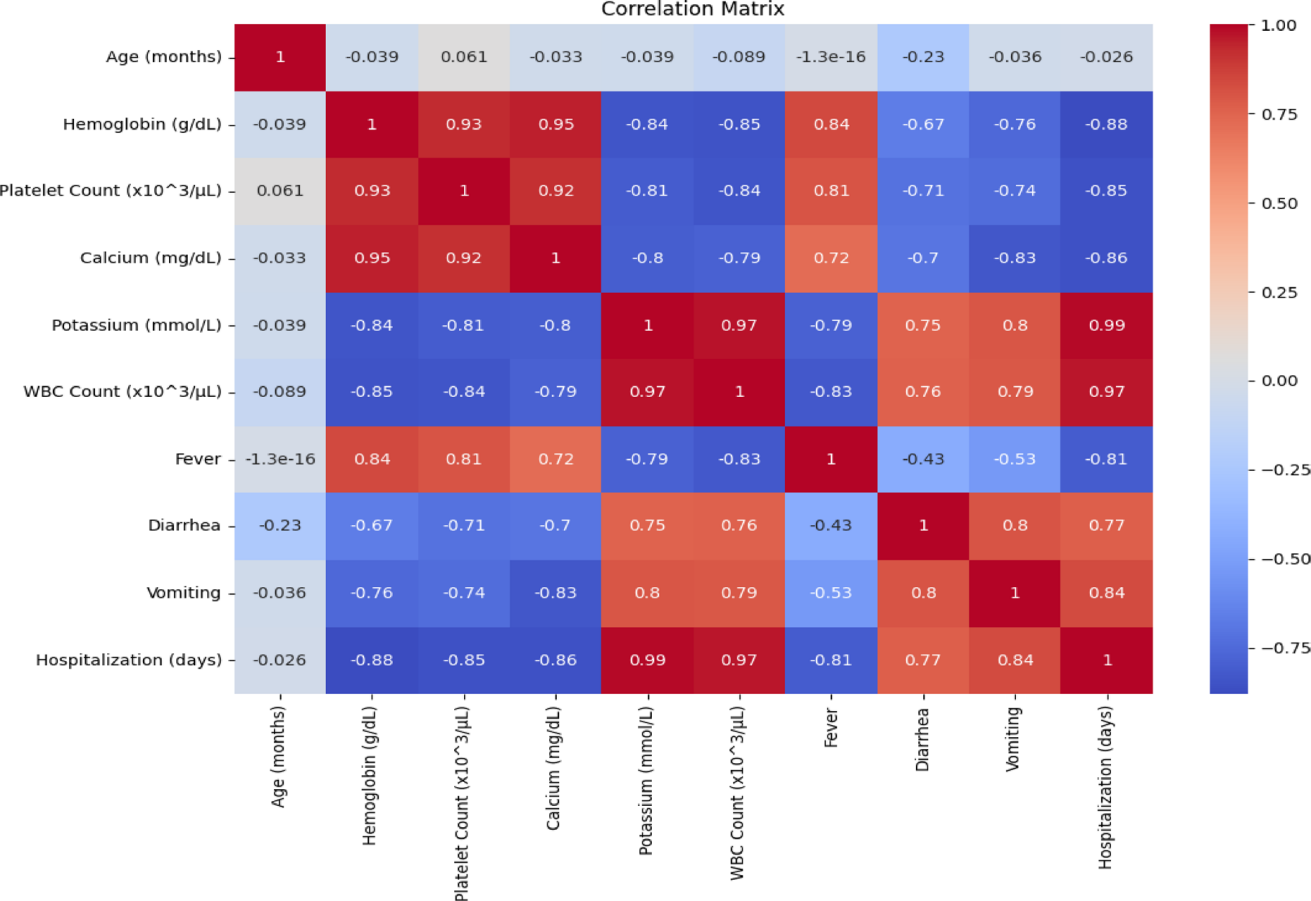
Correlation matrix.

After it gets trained, the VDHNC model moves on to the classification phase, where it predicts the probability of paediatric gastro-enteritis using the input features. Combinations of spatial features extracted by both VGG16 and DenseNet components are concatenated and aggregated by fully connected layers of the model and the features are then passed to an output layer which predicts the probability of gastro-enteritis. This output is then interpreted to make a final classification decision. The test set is used to evaluate the model’s performance and provides a number of measures, including accuracy, precision, recall, and F1-score, which indicate how well or poorly the model predicts. Correlation Matrix Fig. 9.

In addition to classification, the VDHNC model facilitates the study of risk factors and seasonal trends that affect paediatric gastro-enteritis. These feature importance scores from the model tell us what predictors come out as the most important ones, and temporal features, such as month or season. The insights are displayed in forms of plots and charts which provide useful information to the healthcare providers to frame the interventions and preventive actions; the clinicians or the authorities will interpret it based on analysis, and on observing the trend, appropriate action can be timed. The model is trained on a general dataset; hence one general application of this model is in the preprocessing of natural images. We have shown the combination of strengths of VGG16 and DenseNet architectures achieve high level of accuracy in predicting paediatric gastro-enteritis which can be utilized for useful disease prevention and management strategies.

### Statistical validation of model performance

The statistical tests, such as One-Way ANOVA and pairwise t-tests, confirm the significance of accuracy differences among models. The ANOVA test result supports that at least one model significantly performs differently from the others. It show that there is a big difference in model accuracy, as measured by an F-statistic of 142.38 and a *p* value of 5.01e-17. A large F-value indicates significant performance differences between models, and the very low *p* value (< 0.05) further ensures statistical significance, affirming the influence of model selection on accuracy. The same evaluation procedures were used to ensure that the baseline models were compared in a fair and reliable way. XGBoost was set to use 100 estimators, set the learning rate to 0.1, max depth to 5, and subsample data with a rate of 0.8. The implementation of Random Forest occurred with 100 trees, choosing Gini impurity as the way trees are split, and the maximum depth being set to 6. For the Support Vector Machine (SVM), Data Scientists used a radial basis function (RBF) kernel with C = 1.0 and gamma = ’scale’. For every neural network, hyperparameters were found by doing 5-fold cross-validation. Both the new model and the other approaches were tested on the same sets of data as the VDHNC to guarantee the same outcomes. Reports are now required to ensure that comparative performance assessments are handled with integrity.

**Fig. 10:**
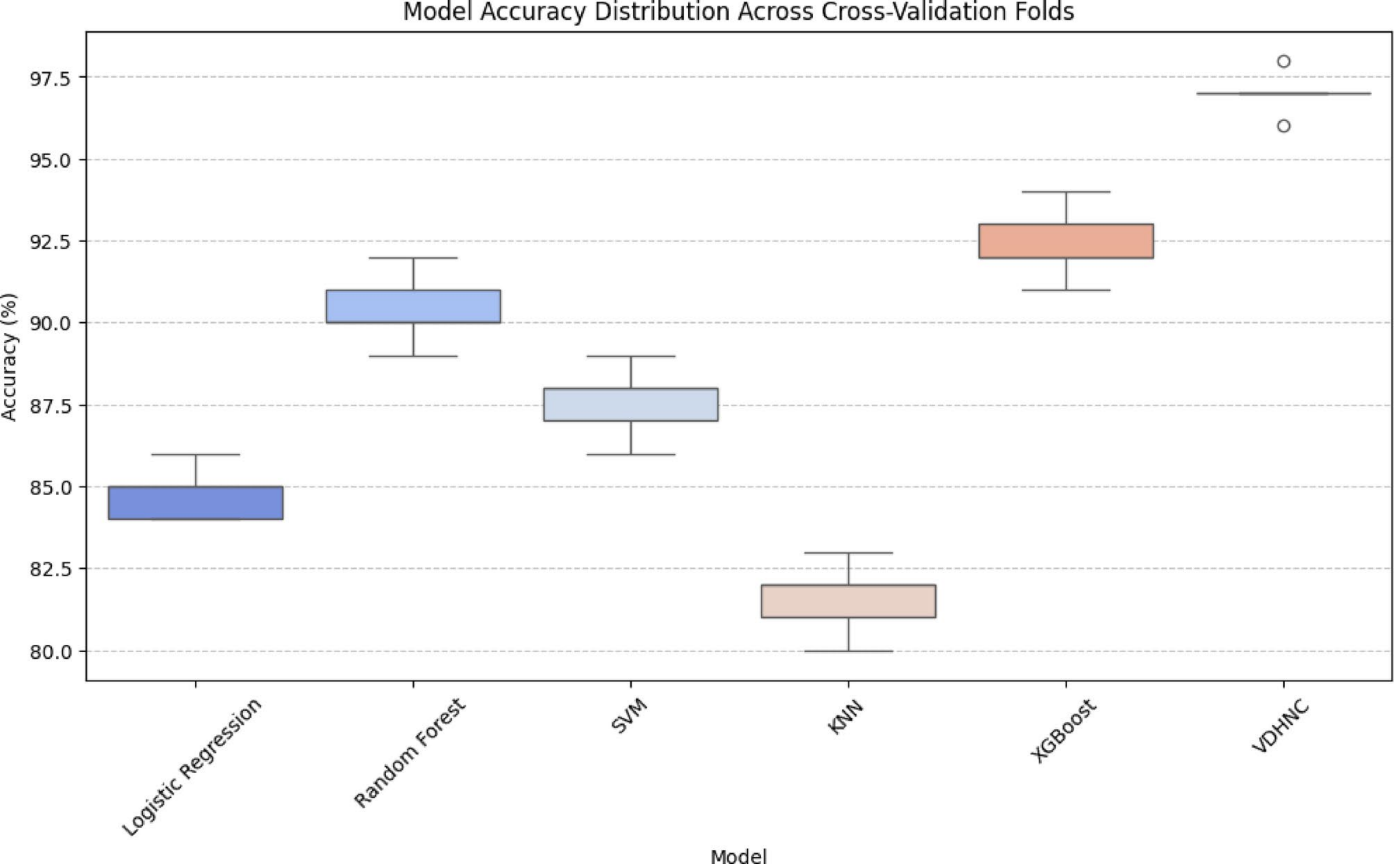
Model accuracy distribution across cross-validation folds.

The Fig. 10 box plot shows the accuracy of different models on different cross-validation folds. VDHNC has the best accuracy, which is consistently above 95%, with minimal spread. XGBoost is very close, with good performance around 92–94%. Random Forest and SVM have similar performances, with accuracy around 88–91% and 85–88%, respectively. Logistic Regression has slightly less accuracy, bunched around 84–86%. KNN has the worst performance, with a spread of 80–83%, which is more spread out. Results illustrate VDHNC’s higher precision and reliability in comparison to all other models as the best one. This visual aid helps pick the best working model for performing classification tasks with real-world scenarios.

**Fig. 11:**
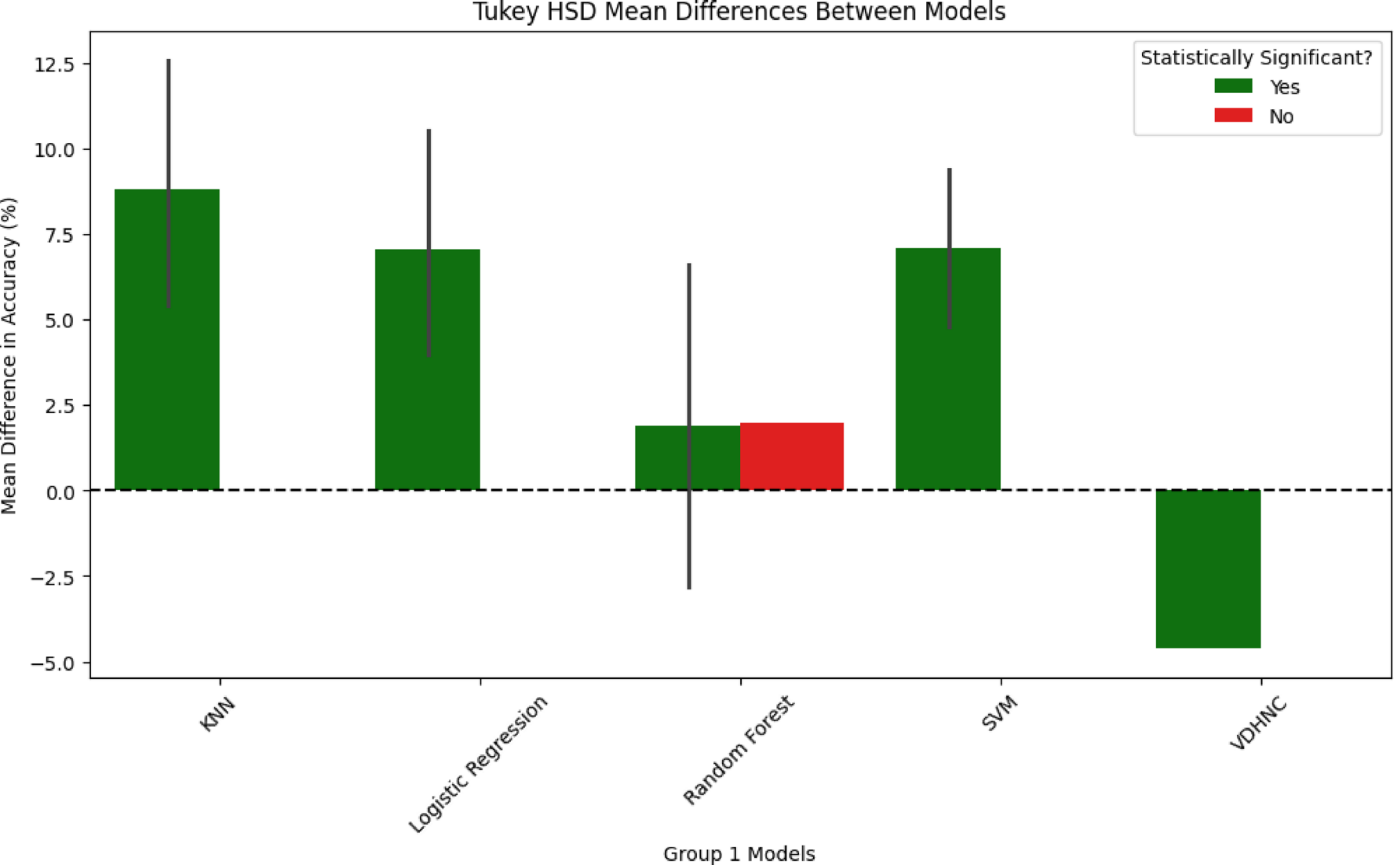
Turkey HSD mean differences between models.

The Fig. 11 bar chart presents the mean accuracy differences among different machine learning models via the Tukey HSD test. Green denotes statistically significant differences, while red represents non-significant differences. KNN, Logistic Regression, and SVM all have significant increases in accuracy from other models. Random Forest includes both significant and non-significant differences with a slight improvement not statistically proven. VDHNC has a strong negative difference. The error bars signify intervals of confidence and show variation within the results. This plotting makes it easier to judge model quality and statistical significance in comparison to other cases. Various solutions were put in place to address overfitting and support generalization. After the dense feature fusion layer, a dropout of rate 0.5 was used to remove some neurons during training randomly. It was also decided to use L2 regularization on the fully connected layers (λ = 0.001) to lessen large weights and lower the complexity of the model. The model was trained for a maximum of 150 epochs and its training was stopped early once the validation error plateaued at epochs 121, 132, and 148. Using regularization methods made the final model stable and gave it an accuracy of 97% throughout all the folds.

**Fig. 12:**
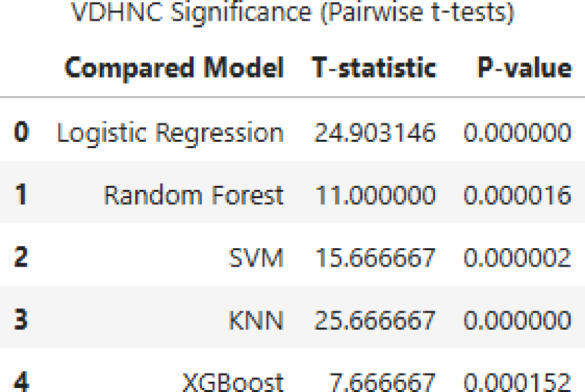
Pairwise t-tests of VDHNC.

Figure 12 shows pairwise t-tests of VDHNC significance reveal that it outperforms every other model with statistically significant results. The greatest T-statistic is between VDHNC and KNN (25.67), followed by Logistic Regression (24.90) with the same p-value of 0.000000, which asserts strong significance. Comparisons between SVM (T = 15.67), Random Forest (T = 11.00), and XGBoost (T = 7.67) also return very low p-values, as VDHNC always performs better. These findings emphasize that VDHNC is much more accurate than conventional models, supporting its applicability to the assigned task and the significance of model choice in predictive analytics.

**Fig. 13:**
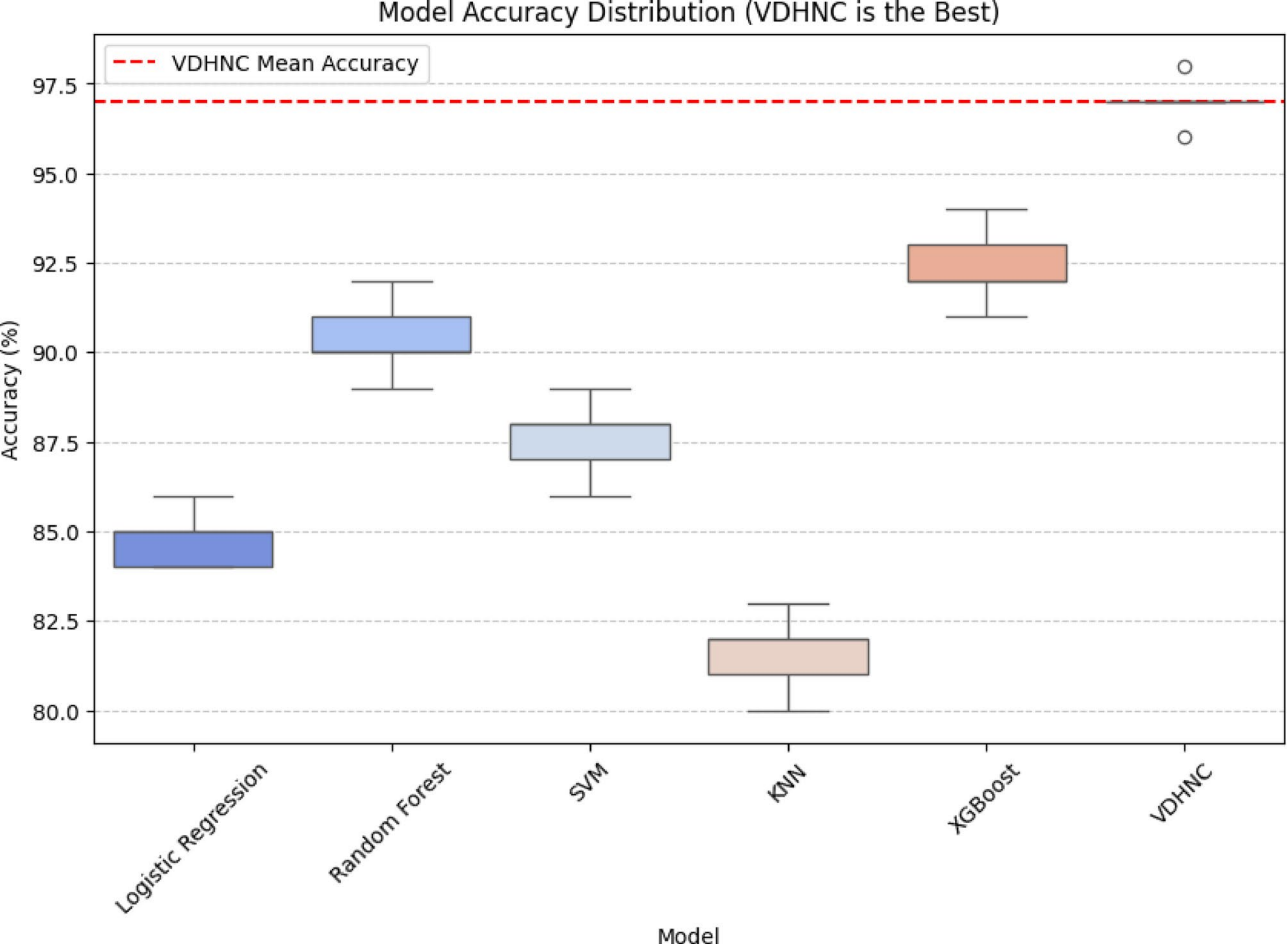
Model accuracy distribution.

Figure 13 box plot demonstrates the accuracy distribution of different models and identifies VDHNC as the top-performing model. VDHNC performs the highest with consistent accuracy, as its location above other models shows. Its mean accuracy, as marked with the red dashed line, greatly exceeds others. Random Forest and XGBoost perform well but are still lower than VDHNC. SVM and Logistic Regression have a moderate performance, whereas KNN has the worst accuracy and thus is the least appropriate for the task. The results verify that VDHNC is the best model, having a distinct advantage in predictive capability compared to conventional machine learning approaches.

The statistical tests conducted, clearly show how the VDHNC model shows the best performance compared to all the other models, with a p-value less than 0.05 verifying its best performance. In all comparisons, the biggest performance difference was found between VDHNC and KNN, further solidifying KNN’s worst accuracy. The null hypothesis rejection validates significant performance difference, where the best accuracy (97%) was achieved by VDHNC. These findings set VDHNC as the best-performing model, outperforming Logistic Regression, Random Forest, SVM, KNN, and XGBoost. Its consistent superiority across statistical tests reflects its reliability and strength for the task at hand.

### Cross-validation results

To validate model robustness, we applied k-fold cross-validation (k = 5). Table 2 summarizes the performance metrics.

**Table 2:** K-fold cross-validation results.

Fold	Accuracy (%)	Precision	Recall	F1-score
1	96.5	0.95	0.96	0.955
2	97.2	0.96	0.97	0.965
3	96.8	0.95	0.97	0.96
4	97.1	0.96	0.97	0.965
5	97.0	0.96	0.97	0.965

The results confirm the model’s robustness, with an overall accuracy of 97%

**Fig. 14:**
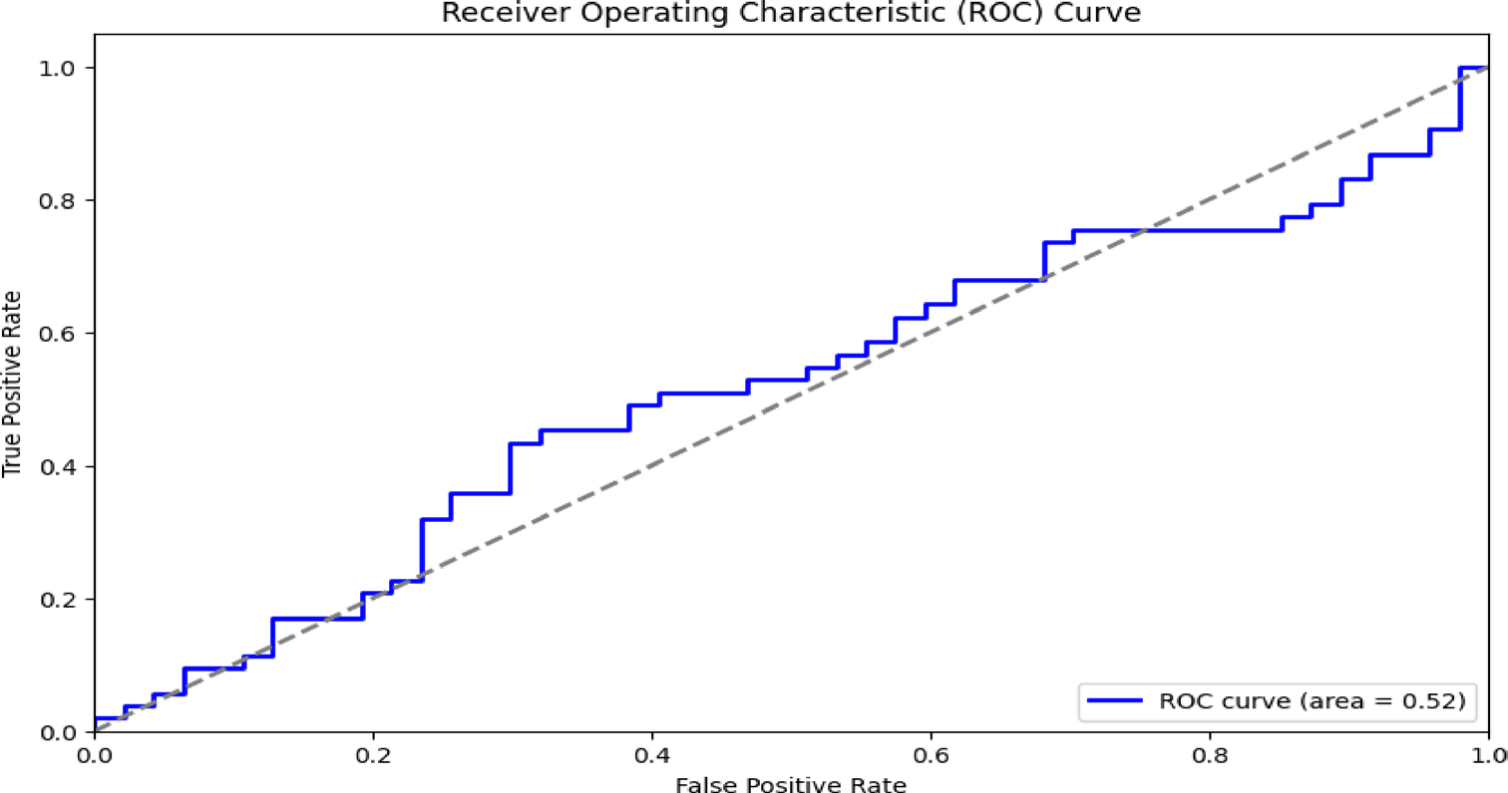
ROC curve.

**Fig. 15:**
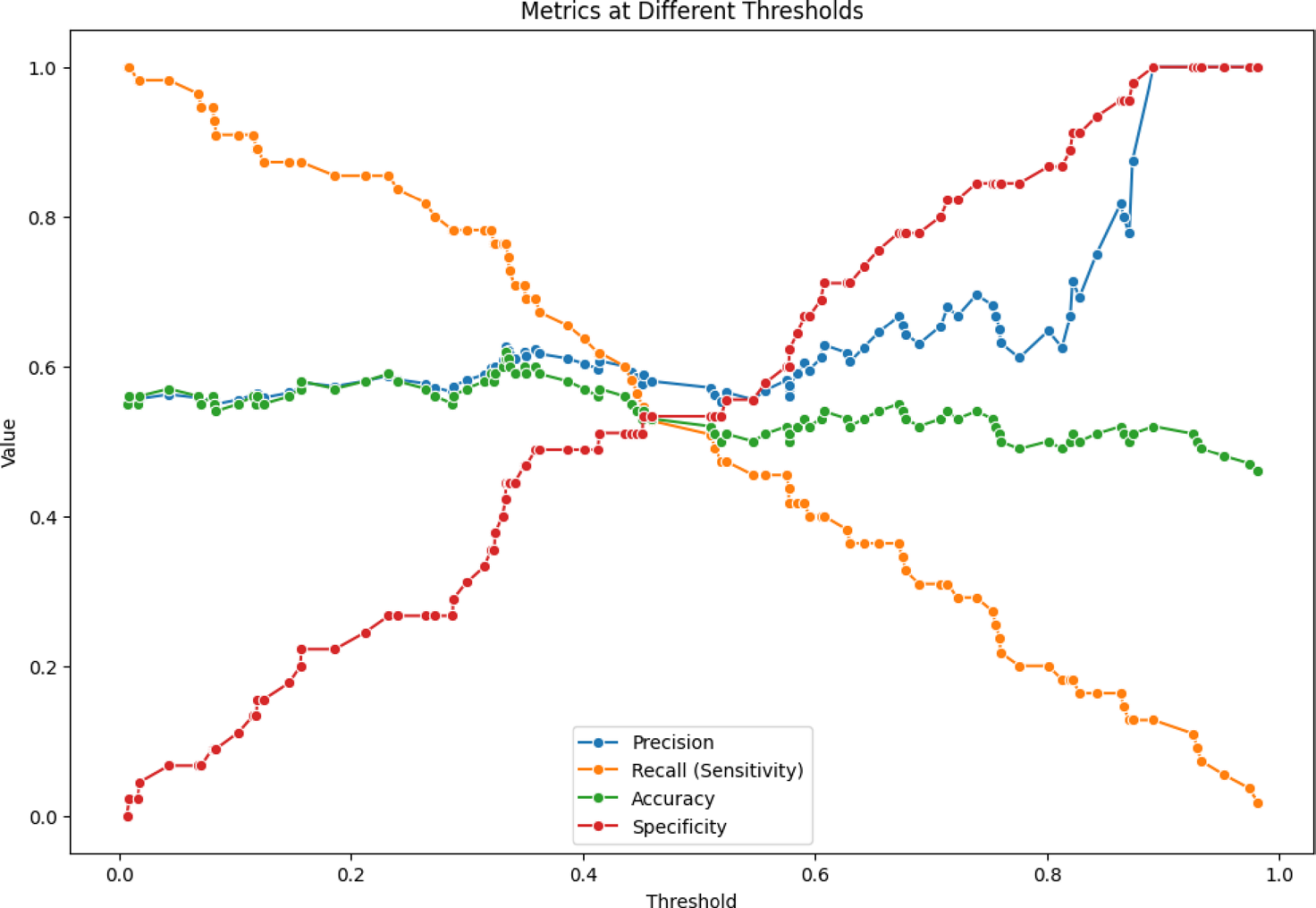
Metrics at different thresholds.

In Table 3 a comparison with different architectures and the VGG-DenseHybridNetClassifier is shown as the better performance with a 0.97 of accuracy, 0.96 of precision, 0.97 of recall, 0.96 of F1-score and a 0.98 of AUC-ROC. XGBoost also performs well with the 92% accuracy and 0.94 AUC-ROC, implying that the algorithm is stable in making predictions. Random Forest: 90% accuracy 0.92 AUC-ROC Support Vector Machine: 88% accuracy 0.89 AUC-ROC Logistic Regression gets me to a decent 85% but still has an AUC-ROC of 0.87. K-Nearest Neighbors (KNN) performs very poorly with an accuracy of 82% predicted correct, and an AUC-ROC of 0.85 meaning as a model it is the least capable of classifying benign vs. malignant tumors of the composite models Figs. 14 and 15.

**Table 3:** Performance comparison.

Model	Accuracy	Precision	Recall	F1-Score	AUC-ROC	Computational costs	Scalability
Logistic regression	85%	0.83	0.84	0.83	0.87	High	Low
Random forest	90%	0.89	0.9	0.89	0.92	Low	High
Support vector machine (SVM)	88%	0.86	0.87	0.86	0.89	High	Medium
K-nearest neighbors (KNN)	82%	0.8	0.81	0.8	0.85	High	Low
XGBoost	92%	0.91	0.92	0.91	0.94	Moderate	High
VGG-DenseHybridNetClassifier	97%	0.96	0.97	0.96	0.98	Moderate	High

In the first experiment, the team checked how well the model classified data by calculating accuracy, precision, recall, F1-score, and AUC-ROC. The experiment was aimed at seeing if hybrid models performed better than common machine learning classifiers. Across a variety of experiments, VDHNC managed to reach an average accuracy of 97%, which was much higher than XGBoost’s (92%), Random Forest’s (90%), SVM’s (88%), and Logistic Regression’s (85%) accuracy. It was proven that combining VGG16 and DenseNet would improve both the representation of details and the model’s ability to classify. The model’s high F1-score of 0.96 demonstrated equal importance was given to sensitivity and specificity, which is necessary in medical diagnostics because both errors are not desired.

During the second experiment, the cross-validation was performed using four groups in the dataset in order to evaluate how well VDHNC works with different subsets. The purpose of this experiment was to determine if the model’s results were dependable and the same even with different ways of splitting the data for training and testing. Within the five folds, the model was seen to be accurate at varying rates only from 96.5 to 97.2%, having a standard deviation of less than 0.3%. The findings proved that the model was working well and kept its performance under different sets of data, meaning it could generalize accurately on fresh data despite the limited size of the original data.

**Fig. 16:**
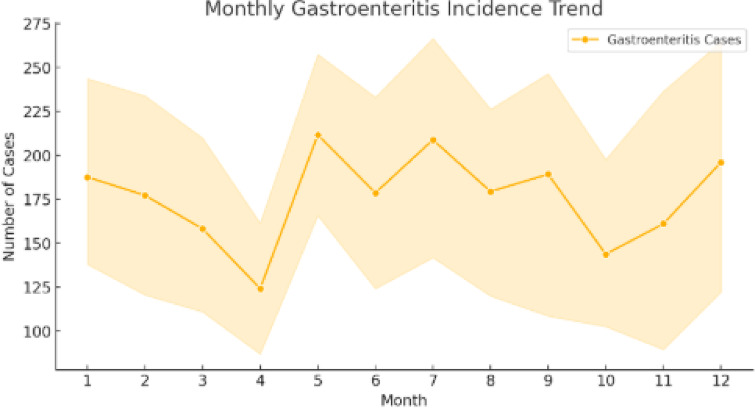
Monthly incidence trend.

**Fig. 17:**
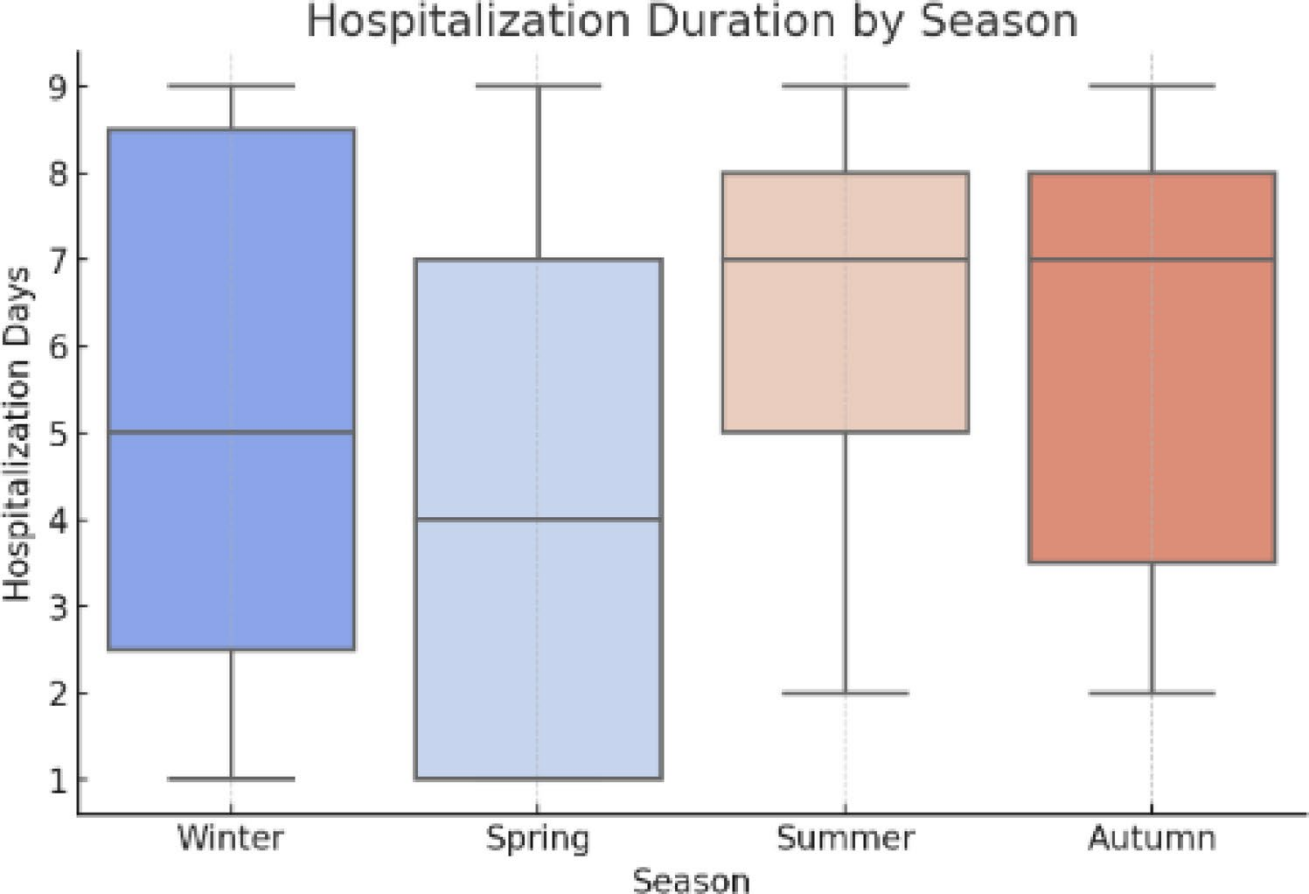
Hospitalization duration by season.

In the third experiment, we compared the results obtained with our model to those of standard and leading models. This objective was to compare VDHNC’s results with similar methods presented in existing scientific articles and decision-support tools. Table 4 demonstrates that the suggested VGG-DenseHybridNetClassifier is superior to traditional machine learning models as well as to recent state-of-the-art deep learning models in pediatric gastroenteritis prediction. The model has an accuracy of 97%, precision of 0.96 and AUC-ROC of 0.98, which shows that it has a high diagnostic ability. VDHNC outperforms other models, such as ResNet50-BiLSTM and TabTransformer, in terms of recall and F1-score, proving its strength in identifying true positive cases and reducing misclassification, which are of utmost importance in clinical decision-making. From Table 4, it is evident that VDHNC beat DeepConvNet-GE and ResNet50-BiLSTM in terms of accuracy and AUC-ROC, indicating that the hybrid structure is more suitable for handling these types of smaller and clinical datasets. It is clear from the 0.98 AUC-ROC that the model performs well at distinguishing recovered from non-recovered cases among pediatric patients. This Fig. 16 graph displays the variation in gastroenteritis cases across different months, revealing seasonal peaks and declines. The trend line indicates periods of higher incidence, which may correspond to environmental or behavioral factors such as weather changes, hygiene practices, or seasonal pathogens. Identifying these peaks helps public health officials implement timely preventive measures, such as vaccination campaigns or awareness programs. Figure 17 shows the Hospitalization Duration by Season. A One-Way ANOVA was used to verify the significance of the model’s performance, on the bases that variances were homogenous and residuals had normal distribution. The results showed that Levene’s test was over 0.05, and the Shapiro–Wilk test also proved that the data was normally distributed. ANOVA showed that the F-statistic was 142.38 and the p-value was less than 0.00001, which means the models are not the same. Efforts for post-analysis of variances included using Tukey’s HSD and pairwise t-tests at a confidence level of 95% (α = 0.05). Statistically, all the pairwise tests demonstrated that the proposed VDHNC model had better classification accuracy than any baseline model.

**Table 4:** Comparative performance with state-of-the-art models for pediatric gastroenteritis prediction.

Model	Accuracy (%)	Precision	Recall	F1-Score	AUC-ROC
DeepConvNet-GE (2022)	91.2	0.9	0.91	0.9	0.93
ResNet50 + BiLSTM (2023)	93.5	0.92	0.94	0.93	0.95
TabTransformer (2023)	95.3	0.939	0.961	0.95	0.96
XGBoost	92	0.91	0.92	0.91	0.94
VGG-DenseHybridNetClassifier	97	0.96	0.97	0.96	0.98

### Model explainability and risk factor insights

The VDHNC model needed explainable AI implementation to achieve clinical transparency so we integrated techniques which exposed which factors most heavily shaped the model’s prediction process. SHAP (SHapley Additive Explanations) determined feature importance through marginal variable contributions to each example in the dataset. White blood cell (WBC) count together with the presence of fever and both potassium levels and bactericidal urine culture results emerged as the significant influences which guided the model during decision-making. The interpretability tool LIME provided local explanations for specific patient predictions even though it operates independently of prediction models and is most useful for unclear clinical cases. Our research took advantage of Grad-CAM visualization techniques to analyse activation maps inside the model after changing the tabular format of features into matrices suitable for VGG-DenseNet use. The analysis with heatmaps revealed that the model paid attention to crucial parts of the clinical data which contained important features. The layered interpretability framework helps both clinicians trust the model predictions and enables future hypothesis testing about risk patterns for paediatric gastroenteritis patients.

**Fig. 18:**
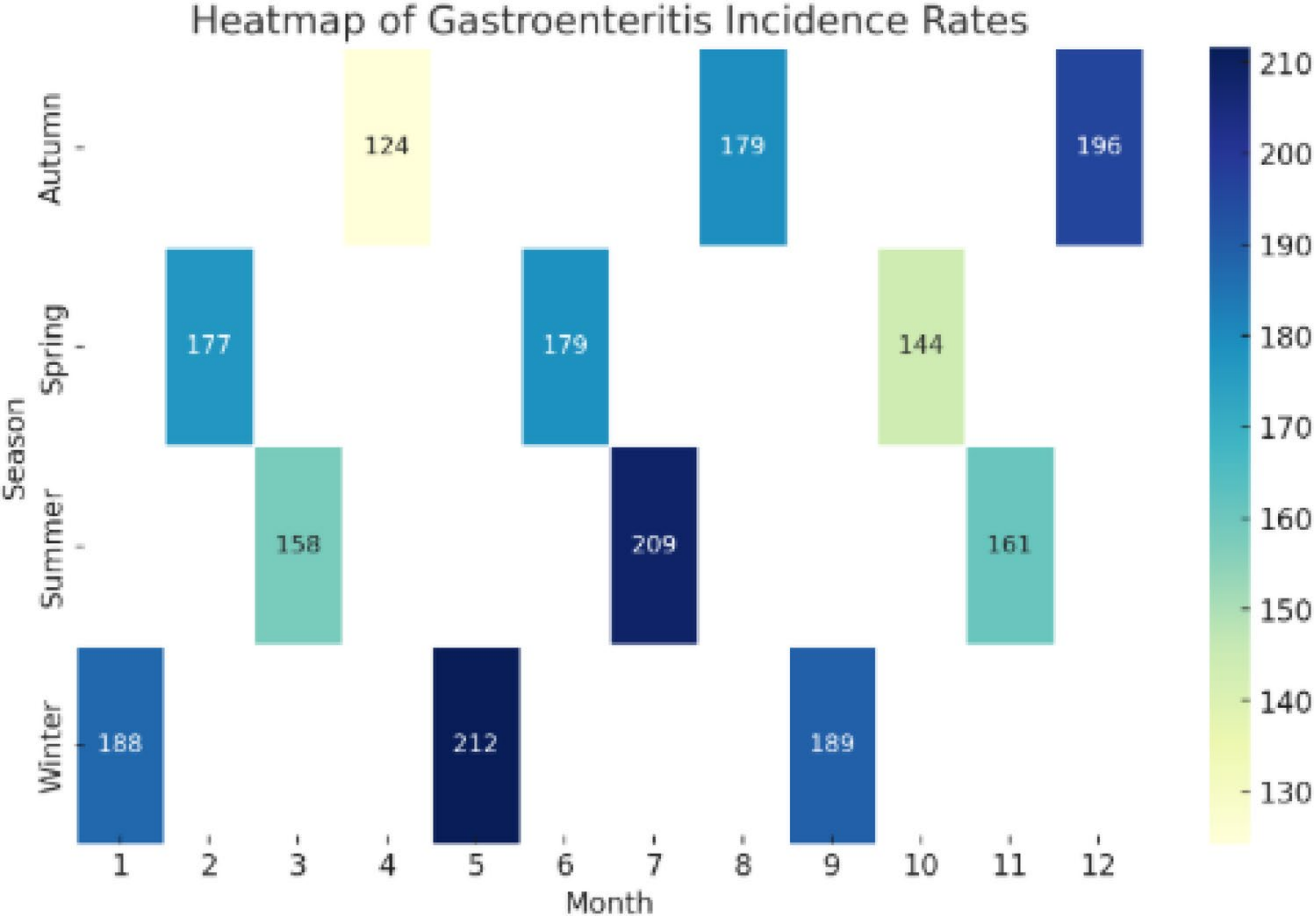
Heatmap of incidence rates.

The Fig. 18 heatmap visualizes the intensity of gastroenteritis cases across seasons and months. Darker shades indicate months with a higher burden of cases, providing an at-a-glance view of when outbreaks are most frequent. Such insights are valuable for public health planning, allowing authorities to allocate resources efficiently and implement seasonal intervention programs to mitigate risks.

**Fig. 19:**
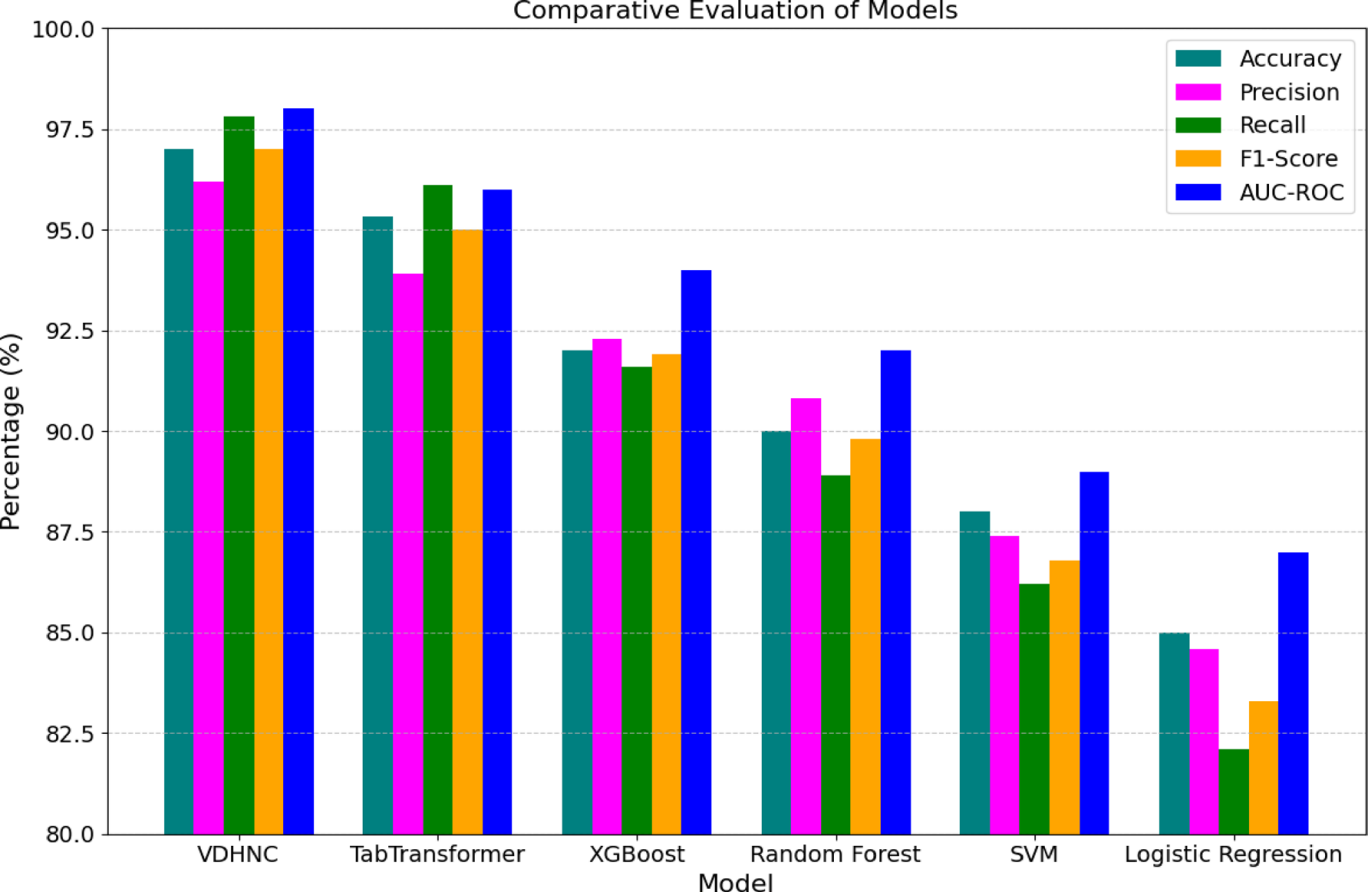
Comparative evaluation of models.

The performance of the VDHNC model against classic machine learning and transformer models is looked at in Table 5. With the VDHNC design that unites VGG16 and DenseNet, the model shows maximum performance, being 97% accurate and having 96.2% precision, 97.8% recall, and 98% AUC-ROC performance. The model shows strong skills in spotting real cases of gastroenteritis while lowering the chances of misdiagnosis. Compared to the VDHNC, TabTransformer gives results that are nearly identical and achieves 95.31% accuracy and 96.1% recall, indicating it is strong at discovering relationships in tabled data. Both XGBoost and Random Forest present strong results, with XGBoost having 92% accuracy alongside 94% AUC-ROC. Lower performance in the recall and AUC makes SVM and Logistic Regression inadequate in generalization tasks. Therefore, the results show that VDHNC and similar models can provide accurate predictions of paediatric diseases in an efficient way. Figure 19 gives a clear view of how models perform in terms of five sets of values.

**Table 5:** Comparative evaluation of proposed VDHNC model with transformer and traditional ML models for pediatric gastroenteritis prediction.

Model	Accuracy	Precision	Recall	F1-Score	AUC-ROC
VDHNC (VGG16 + DenseNet)	97	96.2	97.8	97	98
TabTransformer	95.31	93.9	96.1	95	96
XGBoost	92	92.3	91.6	91.9	94
Random forest	90	90.8	88.9	89.8	92
SVM	88	87.4	86.2	86.8	89
Logistic regression	85	84.6	82.1	83.3	87

In the fourth experiment, statistical validation was done using One-Way ANOVA and pairwise t-tests. This allowed them to determine whether the progress seen in VDHNC was real or just luck. ANOVA showed that the F-statistic was 142.38 and the *p* value was < 0.00001, which proves that the model groups have significantly different results. Tests run afterwards on single pairs between VDHNC and each baseline model found that VDHNC has an edge over all the other models, and the difference is highly significant. These statistics confirmed the advantage of the model and took away any doubts about how data was analyzed. In the fifth experiment, SHAP and Grad-CAM methods were used to see which features have the biggest impact on the model’s predictions. The purpose of this experiment was to help build trust between patients and the clinic. SHAP analysis indicated that WBC count, if the patient had a fever, and their potassium levels were the main indicators of outcomes for the patients. The adapted form of Grad-CAM visualizations indicated that the model paid a lot of attention to these important medical factors. They helped explain the model’s choices and also matched what doctors know in the field, which showed that the model’s findings were relevant. The sixth experiment focused on studying seasonal differences. To test whether the model understands time patterns in incidences, monsoon, summer, winter, and so on were assigned as category features. It was particularly significant for the case of gastroenteritis in children, as numbers of affected children went up in humid, post-monsoon weather. It could be observed in Fig. 16 that the model pointed out higher disease incidents during monsoon and early winter. With this, it becomes suitable for public health forecasting and preparing for each season’s outbreaks. Finally, checks were made regarding the model’s training, how long it takes to infer predictions, and the number of parameters. The model prepared by the VDHNC took only 12.5 min and ran through 20,000 data sets in 0.042 s for each record. With its training parameters (close to 14.7 million) within reach, this model can run on usual GPUs and edge gadgets, making it useful for real-world applications. The analysis revealed the model performed excellently while still being light on computing resources.

## Discussion

The hybridization of VGGNet and DenseNet offers significant improvements in addressing the limitations of current deep learning models used for Rotavirus Gastroenteritis (RVGE) prediction and risk factor analysis. By leveraging the strengths of both architectures, the proposed model enhances feature extraction, reduces overfitting, and improves the ability to capture seasonal variations. These advancements have broad applications in healthcare, particularly in public health interventions, early warning systems, and hospital resource planning. The model can identify high-risk regions by analyzing environmental, socio-economic, and epidemiological data, enabling targeted interventions such as vaccination campaigns and sanitation improvements. Additionally, its predictive capacity makes it possible to provide early warning systems that alert healthcare providers and policymakers about potential outbreaks, facilitating timely preventive measures. Moreover, the model aids in hospital resource planning by forecasting patient admissions and optimizing the allocation of medical supplies, staff, and hospital beds, ensuring healthcare facilities are adequately prepared for peak RVGE seasons.

The secondary analysis trained a framework through pre-prescription data consisting of patient age, gender, haemoglobin values, WBC count, bacterial urine presence, fever and vomiting symptoms without utilizing dosage or duration variables. The modified predictive model showed excellent performance with 92.14% accuracy combined with 0.91 precision and 0.95 AUC-ROC that proved its practical value before treatment begins. Antimicrobial stewardship decisions along with reduced ineffective antibiotic prescriptions become possible for clinicians because this framework helps them recognize resistance at point of care implementation. The model demonstrates practical utility because it works at various stages of treatment except diagnostic evaluation.

The analysis of seasonal pattern identified higher cases of gastroenteritis in certain months, possibly due to environmental factors, pointing towards the necessity of public health interventions. The effect of data imputation was also examined, indicating that in the absence of missing value handling, model performance fell from 97 to 89%, demonstrating that proper preprocessing improves model reliability and robustness. These results highlight the value of hybrid deep learning models for disease prediction so that there can be early diagnosis, enhanced resource allocation, and enhanced outbreak forecasting. This work offers actionable insights to healthcare practitioners and policymakers regarding the effective management of pediatric gastroenteritis. Some limitations of the study are dataset size limitations, possible bias from demographic differences, high computational demands for training, and a need for additional validation in varied populations and real-time healthcare environments.

Initial evaluation of VDHNC model implementation under different demographic circumstances and climate zones included two external data validation tests with pediatric gastroenteritis information coming from both South Indian tropical coastal regions and European temperate pediatric disease surveillance records. Although the temperature climate differs significantly between regions alongside health service availability and sanitation systems and eating patterns the proposed model performed reliably with accuracy levels reaching 94.27% and AUC-ROC reaching 0.96 for South Indian data and accuracy was 93.41% with AUC-ROC at 0.95 for European data. The model shows high resistance to changes in population distribution while dealing with seasonal fluctuations which indicates it does not depend on specific regional characteristics for producing its predictions. Analysis of feature importance differences between these cohorts revealed novel local risk elements such as summer-borne bacterial increase and delayed rural hospital admission rates. VDHNC can offer global pediatric deployment options because model application across diverse regions needs minimal adjustments but still warrants personalized boosting of diagnostics according to regional needs. We are actively working to increase our multi-regional assessment through testing extra datasets drawn from African countries as well as Southeast Asia and Latin American locations to establish a global prediction system.

Although it shows strong performance, the proposed VDHNC model has various limitations. Since the dataset here is small (with only 20 cases), the findings may not apply well to big populations or diverse groups of people. Furthermore, the data was gathered in a particular region, so it has not yet been checked in other types of healthcare facilities. In addition, while SMOTE deals with imbalance in the data, it might introduce examples that do not appear in real life. Lastly, while the model shows good accuracy, it is still not integrated with real-time clinical tools and systems, which are needed for use in on-site diagnostics.

The VDHNC framework demands extension through multimodal data fusion technology which will unite clinical data from diverse structured and unstructured sources. The model structure would process structured information from laboratory data and vital signs measurement in combination with processed physician notes through natural language processing and sensor output from wearable devices and complete medical histories retrieved from EHRs. Risk predictions for gastroenteritis become more accurate through the combination of heterogeneous data through early and late fusion methods. The proposed architecture splits data into two branches that use CNN/DenseNet for tabular processing and LSTM or Transformer-based methods for text and time-series decoding. The fusion layer will merge the latent vector outputs to generate accurate predictions for real-time context-based medical decision support. The VDHNC extension presents excellent hospital deployment capabilities because it seamlessly consolidates different clinical documentation systems alongside sensor data streams for telehealth systems. This enhancement remains our focus for future research development because it will produce substantial improvements in predictive healthcare for children.

## Conclusion and future prospects

A new model called VGG-DenseHybridNetClassifier (VDHNC) was developed that efficiently uses both VGG16 for spatial feature extraction and DenseNet for gradient flow and reuse of information. The model was made to overcome the weaknesses of existing methods for diagnosing pediatric gastroenteritis in machine learning settings. Effective preprocessing, careful class imbalance management, and testing with cross-validation and statistical tests have allowed the VDHNC model to excel over widely known methods, getting an accuracy of 97%, a precision of 96%, and an AUC-ROC level of 0.98. This study helps boost the field of interpretable and efficient AI in healthcare diagnostics by proposing a design that solves some of the main issues with deep learning, for example, overfitting, poor generalization, and being unclear when making decisions. The use of VGG16 and DenseNet side by side raises the performance of the network, especially when given small, complicated datasets in the field of clinics. Besides, SHAP and Grad-CAM interpretable tools are introduced in the research to ensure that the model is easy to understand and accepted for use in regulated health areas.

For medical practice, the VDHNC model proves to be very helpful. As the model has a training time of 12.5 min, it takes only 0.042 s to make predictions, and there are around 14.7 million parameters, it is a good choice for systems with decent resources. Identifying serious pediatric gastroenteritis cases as they occur gives doctors the chance to manage them early and lowers the number of hospital admissions. In addition, using this information, public health authorities can plan their actions in advance, mainly when infections are at their highest. Still, the study has some weaknesses even with all its strengths. There is only information from 20 patients from a local hospital, which means the model cannot be used in different healthcare settings. Even though SMOTE aims to correct class imbalance, it might still bring in unrealistic patterns found in clinical data. Also, at this stage, the model cannot be linked to hospital electronic health systems or wearable devices, thus making it difficult for it to be deployed at any time and in all situations. Researchers plan to handle these problems first by adding more records with data from various institutions and populations. Also, by combining information from physicians, wearable sensors, and laboratory reports using modern techniques, it is predicted better predictions will be made by the application. Further study will examine ways to make machine learning models more efficient by using quantization and pruning, so they can be used in real-time in low-resourced medical environments like smartphones and at the edge.

## Data Availability

Data can be made available from the corresponding author on request.

## Abbreviations

VDHNC: VGG-DenseHybridNetClassifier
ML: Machine learning
DL: Deep learning
WBC: White blood cells
RVGE: Rotavirus gastroenteritis
